# Ligand‐Free Copper‐Catalyzed Ullmann‐Type C−O Bond Formation in Non‐Innocent Deep Eutectic Solvents under Aerobic Conditions

**DOI:** 10.1002/cssc.202102211

**Published:** 2021-12-06

**Authors:** Andrea Francesca Quivelli, Manuela Marinò, Paola Vitale, Joaquín García‐Álvarez, Filippo M. Perna, Vito Capriati

**Affiliations:** ^1^ Dipartimento di Farmacia – Scienze del Farmaco Università di Bari “Aldo Moro” Consorzio C.I.N.M.P.I.S. Via E. Orabona 4 I-70125 Bari Italy; ^2^ Laboratorio de Química Sintética Sostenible (QuimSinSos) Departamento de Química Orgánica e Inorgánica (IUQOEM) Centro de Innovación en Química Avanzada (ORFEO-CINQA) Universidad de Oviedo 33071 Oviedo Spain

**Keywords:** copper, cross-coupling, deep eutectic solvents, ethers, sustainable chemistry

## Abstract

An efficient and novel protocol was developed for a Cu‐catalyzed Ullmann‐type aryl alkyl ether synthesis by reacting various (hetero)aryl halides (Cl, Br, I) with alcohols as active components of environmentally benign choline chloride‐based eutectic mixtures. Under optimized conditions, the reaction proceeded under mild conditions (80 °C) in air, in the absence of additional ligands, with a catalyst [Cu^I^ or Cu^II^ species] loading up to 5 mol% and K_2_CO_3_ as the base, providing the desired aryloxy derivatives in up to 98 % yield. The potential application of the methodology was demonstrated in the valorization of cheap, easily available, and naturally occurring polyols (e. g., glycerol) for the synthesis of some pharmacologically active aryloxypropanediols (Guaiphenesin, Mephenesin, and Chlorphenesin) on a 2 g scale in 70–96 % yield. Catalyst, base, and deep eutectic solvent could easily and successfully be recycled up to seven times with an E‐factor as low as 5.76.

## Graphical abstract

**Inside is better than out**: Aryl alkyl ethers and valuable active pharmaceutical ingredients are obtained via a Cu‐catalyzed Ullmann‐type cross‐coupling reaction between (hetero)aryl halides and naturally occurring alcohols as active components of environmentally friendly choline chloride‐based eutectic mixtures, under hydrous and mild conditions, in the absence of ligands, and with an effective recycling of eutectic mixture, catalyst, and base.

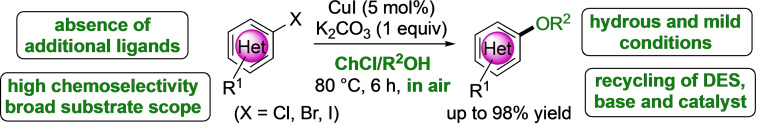

## Introduction

Aryl ethers are versatile synthetic intermediates for the preparation of polymeric materials, find applications in chemical engineering, pharmaceuticals, food, and agrochemicals, and are also found in several biologically active compounds.^[1]^ Traditional approaches to access aryl ethers mainly rely on nucleophilic aromatic substitution or Williamson and Mitsunobu reactions. Complementary approaches are based on copper‐ or metal‐mediated C−O bond forming reactions.[^2^, ^3^] The use of strongly coordinated/electron‐rich ligands, together with volatile organic compounds (VOCs) and harsh reaction conditions, are common features of the above‐mentioned synthetic routes. The Ullmann ether synthesis has also been limited by low to moderate yields in the final adducts.^[3d]^

**Scheme 1:**
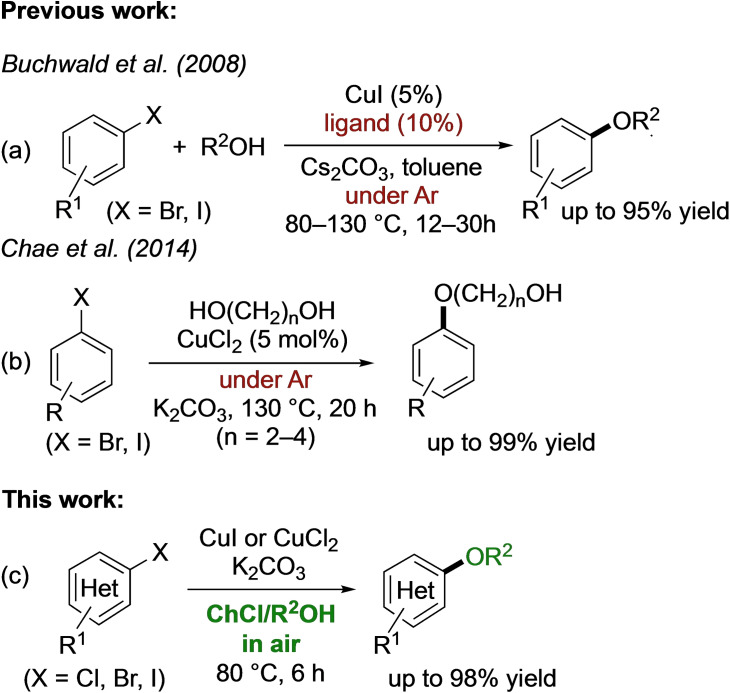
(a) Ligand‐based Cu^I^‐catalyzed C−O coupling reactions of aryl halides with alcohols in toluene under Ar.^[9]^ (b) Ligand‐free Cu^II^‐catalyzed C−O coupling reactions of aryl halides with aliphatic diols under Ar.^[10]^ (c) Unprecedented ligand‐free Cu^I^‐ or Cu^II^‐catalyzed C−O coupling reactions between aryl halides and alcohol‐based DESs in air.

Although, in the last two decades, the copper‐catalyzed preparation of diaryl ethers has benefited a lot in terms of reaction rate from the introduction of ligands such as amino acids,^[4]^ hydroxyquinolines,^[5]^ diones,^[6]^ Schiff bases,^[7]^ and well‐defined Cu^I^ complexes,^[8]^ the synthesis of aryl alkyl ethers has remained largely unexplored. Buchwald and co‐workers first reported the successful use of phenanthroline ligands to promote the copper‐catalyzed C−O cross‐coupling reaction between aliphatic alcohols and aryl iodides and bromides, working in toluene at 80–130 °C for 12–30 h, under an Ar atmosphere (Scheme 1a).^[9]^ In 2014, Chae and co‐workers^[10]^ described an efficient Cu^II^‐catalyzed C−O coupling between aryl bromides and iodides and aliphatic diols (which acted as reactants, ligands, and solvents) to generate the corresponding hydroxyalkyl aryl ethers, when working at 130 °C for 20 h, under an Ar atmosphere (Scheme 1b). As part of our ongoing research program on the synthesis of heterocycles^[11]^ and on bio‐,^[12]^ metal‐catalyzed, and metal‐mediated organic transformations^[13]^ run in environmentally responsible solvents like water and deep eutectic solvents (DESs),^[14]^ we recently introduced a general methodology for the direct Cu^I^‐catalyzed C−N coupling reactions between aliphatic/aromatic amines^[15a]^ or primary/secondary amides^[15b]^ and (hetero)aromatic halides, taking place in a choline chloride/glycerol (ChCl/Gly)^[15a]^ or in a ChCl/water (or even in water)^[15b]^ eutectic mixture, under mild and bench‐type reaction conditions.

Herein, we describe a sustainable copper‐catalyzed Ullmann‐type ether synthesis in a ChCl‐based DES including an alcohol component, which also acts as a reagent. Notably, the cross‐coupling proceeds: (a) under mild conditions (80 °C) in air; (b) in the absence of additional ligands; (c) with a broad substrate scope encompassing an array of (hetero)aryl bromides, iodides, and even chlorides; (d) with a catalyst loading of Cu^I^ or Cu^II^ up to 5 mol%; (e) with an effective recycling of the eutectic mixture, the catalyst, and the base; and (f) with the desired aryl alkyl ethers isolated in up to 98 % yield (Scheme 1c). Furthermore, the versatility of the reported protocol was displayed by targeting three pharmacologically active aryloxypropanediols (Guaiphenesin, Mephenesin, and Chlorphenesin) on a 2 g scale.

## Results and Discussion

We initiated our investigation by reacting bromobenzene **1** **a** (1.0 mmol; chosen as model substrate) with the eutectic mixture ChCl/Gly (1 : 2 mol mol^−1^; 1.0 g), under the same conditions previously used for the coupling of aliphatic amines with aryl halides, that is, in the presence of CuI (10 mol%) as the catalyst and K_2_CO_3_ (2 equiv.) as the base.^[15a]^ While no reaction took place at 60 °C, we were pleased to find that upon increasing the temperature to 80 °C, substrate **1** **a** underwent complete conversion, although characterized by slow reaction rate (6 h) (see the Supporting Information), to give an inseparable mixture of two isomeric adducts resulting from the coupling of the primary (**2** **a**) and secondary (**3** **a**) alcohol function of Gly with **1** **a** (**2** **a**/**3** **a**: 77 : 23) (Table 1, entries 1 and 2). Similar results were obtained by halving the amount of both CuI (5 mol%) and K_2_CO_3_ (1 equiv.), with the final ratio of **2** **a**/**3** **a** being 80 : 20 (Table 1, entry 3). Iodobenzene (**1** **b**) displayed similar reactivity, whereas chlorobenzene (**1** **c**) reacted slower, providing a mixture of **2** **a** and **3** **a** in 30 % yield only after 24 h reaction time at 100 °C (Table 1, entries 4 and 5). No coupling was observed by running the reaction in the absence of base or CuI at 80–100 °C, whereas a screening of bases revealed that both Cs_2_CO_3_ and *t*BuOK were equally effective at 80 °C (Table 1, entries 6–9). Interestingly, in contrast to what was observed in Cu‐catalyzed coupling reactions of aryl bromides with diols with no additional solvents,^[10]^ in this work, both Cu^I^ and Cu^II^ salts exhibited the same reactivity, thereby furnishing the adducts **2** **a**/**3** **a** in almost quantitative yield (90–98 %) after 6 h, most probably because of a similar solubilization in or stabilization by the employed eutectic mixture (Table 1, entries 10 and 11). This is an important point because Cu^II^ salts are known to be cheaper and characterized by higher air stability and water solubility, which simplifies the work‐up procedure. Most authors agree that Cu^I^ is the true catalytic species, with Cu^II^ being in‐situ reduced to Cu^I^ by the excess O‐nucleophile in the presence of a base.^[16]^

**Table 1:** Optimization of Ullmann‐type C−O bond formation between halobenzene **1** and Gly of the corresponding eutectic mixture to give adducts **2** **a** and **3** **a**.^[a]^

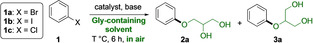						
						
Entry	Solvent	Catalyst (mol %)	Base (equiv.)	*T* [°C]	**2a**+**3a** yield^[b]^ [%]	**2a**/**3a** ^[c]^
1	ChCl/Gly^[d]^	CuI (10)	K_2_CO_3_ (2)	60	–^[e]^	–
2	ChCl/Gly^[d]^	CuI (10)	K_2_CO_3_ (2)	80	98	77 : 23
3	ChCl/Gly^[d]^	CuI (5)	K_2_CO_3_ (1)	80	98	80 : 20
4	ChCl/Gly^[f]^	CuI (5)	K_2_CO_3_ (1)	80	98	80 : 20
5	ChCl/Gly^[g]^	CuI (5)	K_2_CO_3_ (1)	100	30^[e]^	80 : 20
6	ChCl/Gly^[d]^	CuI (5)	–	80	NR^[h]^	–
7	ChCl/Gly^[d]^	–	K_2_CO_3_ (1)	100	NR^[h]^	–
8	ChCl/Gly^[d]^	CuI (5)	Cs_2_CO_3_ (1)	80	98	78 : 22
9	ChCl/Gly^[d]^	CuI (5)	*t*BuOK (1)	80	98	77 : 23
10	ChCl/Gly^[d]^	CuO (5)	K_2_CO_3_ (1)	80	90	78 : 22
11	ChCl/Gly^[d]^	CuCl_2_ (5)	K_2_CO_3_ (1)	80	98	80 : 20
12	ChCl/Gly^[d]^	Pd(OAc)_2_ (5)	K_2_CO_3_ (1)	100	–^[i]^	–
13	Gly	CuI (5)	K_2_CO_3_ (1)	100	75	76 : 24
14	ChCl/Gly^[j]^	CuI (5)	K_2_CO_3_ (1)	80	96	80 : 20
15	ChCl/Gly^[k]^	CuI (5)	K_2_CO_3_ (1)	80	98	80 : 20
16	Pro/Gly	CuI (5)	K_2_CO_3_ (1)	100	–	–
17	betaine/Gly	CuI (5)	K_2_CO_3_ (1)	100	20^[l]^	78 : 22

[a] Reaction conditions: 1.0 g DES or 1 mL Gly per 1.0 mmol of **1** **a**–**c**; DES: ChCl/Gly (1 : 2, 1 : 1, or 1 : 3 mol mol^−1^); l‐proline (Pro)/Gly (2 : 5 mol mol^−1^); betaine/Gly (1 : 2 mol mol^−1^). [b] The yields reported are for products isolated and purified by column chromatography. [c] Calculated by ^1^H NMR spectroscopy of the crude reaction mixture using an internal standard technique (NMR internal standard: CH_2_Br_2_). [d] X=Br. [e] Reaction time: 24 h. [f] X=I. [g] X=Cl. [h] NR=no reaction. [i] Biphenyl was the only adduct isolated (98 % yield). [j] 1 : 1 mol mol^−1^. [k] 1 : 3 mol mol^−1^. [l] After 24 h: 55 % yield.

It is also worth mentioning that this unprecedented methodology is able to valorize glycerol, a natural polyol produced as the main by‐product in the biodiesel industry and in the conversion of cellulose and lignocellulose, for the preparation of high added‐value products (see below).^[17]^

**Scheme 2:**
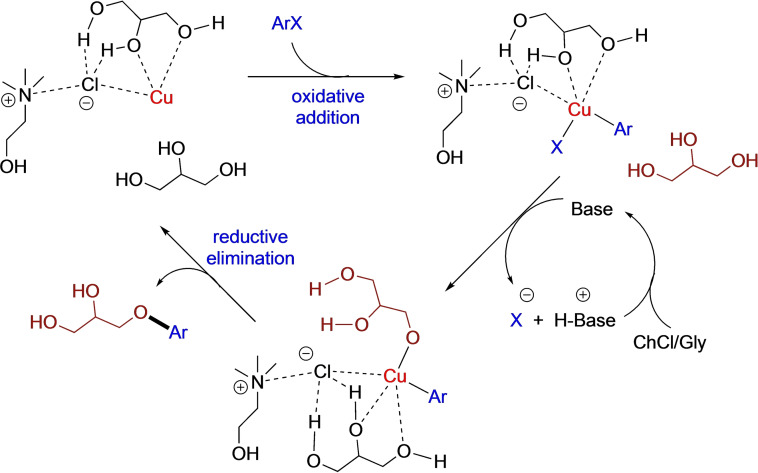
Possible catalytic cycle for the Ullmann C−O coupling reaction promoted by ChCl/Gly.

By replacing Cu with a Pd catalyst [e. g., Pd(OAc)_2_], the homocoupling product of bromobenzene (biphenyl) was the only adduct isolated in 98 % yield (Table 1, entry 12), while by changing the solvent to pure Gly the overall yield of **2** **a**/**3** **a** dropped down to 75 %, the remainder being starting material only (Table 1, entry 13). This result is consistent with a positive, stabilizing effect exerted by both the DES components (ChCl and Gly) on the copper salt, eventually leading to an improved catalytic performance for C−O coupling reaction (Scheme 2). It is worth noting that ligandless C−O bond formation reactions in DESs, aimed at synthesizing α‐acyloxy carbonyl compounds and diaryl ethers, have also been found to take place via oxidative‐coupling pathways^[18a]^ and by exploiting a Fe_3_O_4_@creatine Cu^I^ magnetic catalyst,^[18b]^ respectively.

By varying the molar ratio between ChCl and Gly (1 : 1 or 1 : 3), the new eutectic mixtures proved to be similarly effective in promoting the coupling reaction (overall yield: 96–98 %; Table 1, entries 14 and 15). On the other hand, the reaction yield was zero or up to 20 % when alternatively using l‐proline (Pro) or betaine, respectively, as the hydrogen bond acceptor in combination with Gly (Table 1, entries 16 and 17). It is worth mentioning that the direct coupling of **1** **a** or **1** **b** with external alcohols [e. g., ethylene glycol (EG), 2‐butanol, benzylic alcohol] in various hydrophilic and hydrophobic DESs [e. g., ChCl/urea (1 : 2 mol mol^−1^), decanoic acid/menthol (1 : 2 mol mol^−1^), acetic acid/menthol (1 : 1 mol mol^−1^)] and under different reaction conditions in terms of bases (e. g., *t*BuOK, Cs_2_CO_3_, CH_3_CO_2_Na, K_2_CO_3_), catalysts [CuI, CuCl_2_, CuO, Pd(OAc)_2_ 5–20 mol%], temperatures (up to 130 °C), reaction times (up to 48 h), and additional ligands (e. g., 1,10‐phenantroline, l‐proline, *N*,*N*‐dimethylglycine, ethylenediamine) proved to be totally ineffective (see the Supporting Information). When using a sugar‐based DES, a complex mixture of regioisomers was obtained, which was not further investigated (see the Supporting Information). Overall, these data are also consistent with the fact that the presence in the eutectic mixture of components with more than one oxygen‐containing functional groups seems to be a prerequisite to trigger C−O bond formation reactions through copper chelation, with DES behaving as a ligand (Scheme 2).

**Scheme 3:**
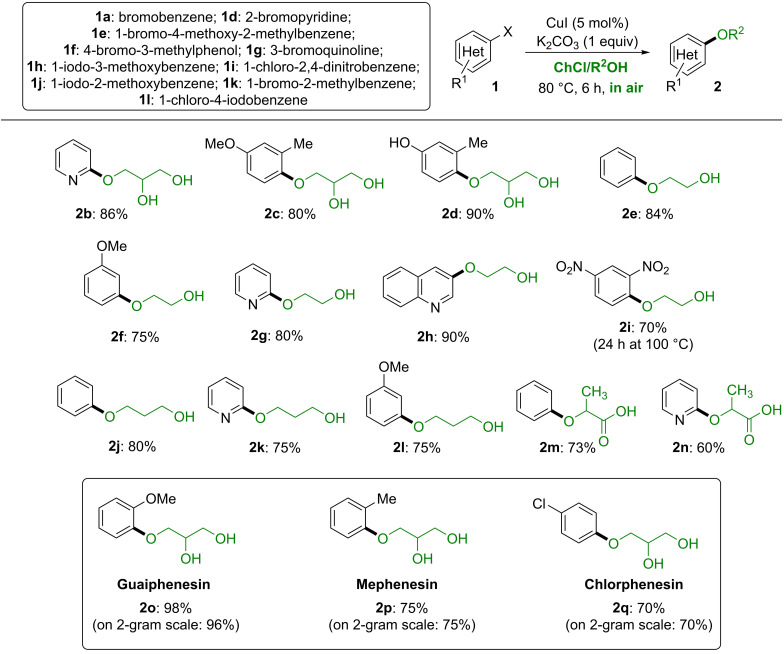
Synthesis of aryl alkyl ethers **2** via copper‐catalyzed cross‐coupling reactions of (hetero)aryl halides **1** (1 mmol) with alcohols of ChCl‐based eutectic mixtures (1 g). The yields reported are for products isolated and purified by column chromatography. Products **2** **b**–**d** and **2** **o**–**q** from DES ChCl/Gly (1 : 2 mol mol^−1^). Products **2** **e**–**i** from DES ChCl/ethylene glycol (1 : 2 mol mol^−1^). Products **2** **j**–**l**, from DES ChCl/1,3‐propanediol (1 : 2 mol mol^−1^). Products **2** **m**,**n** from DES ChCl/L‐lactic acid (1 : 2 mol mol^−1^). In the case of products **2** **b**,**c**,**j**,**m**–**q**, the same yield was obtained when using CuCl_2_ as the catalyst.

With optimized conditions identified as ChCl/Gly (1 : 2 mol mol^−1^), CuI or CuCl_2_ (5 mol%), K_2_CO_3_ (1 equiv.), 80 °C, 6 h, in air, we investigated the generality of this transformation by reacting several (hetero)aryl halides with alcohol‐containing ChCl‐based eutectic mixtures (Scheme 3). The reaction of an electron‐poor heterocycle like 2‐bromopyridine (**1** **d**) or electron‐rich alkyl‐ and alkoxy‐substituted bromo arenes in *ortho*‐ and *para*‐position like **1** **e**,**f** with ChCl/Gly afforded adducts **2** **b**–**d** with high chemoselectivity in 80–90 % yield. The products of coupling of the secondary alcohol function at C2 in Gly formed in no more than 9 % when synthesizing **2** **c** or in traces in the case of **2** **d**, but they could easily be separated. It is also worth mentioning that, under these conditions, the coupling products deriving from the involvement of the two primary functions of glycerol at C1 and C3 (1,3‐diaryloxypropan‐2‐ols) were never observed. At this point, we would like to highlight that our catalytic system is able to tolerate the presence of good *N*‐donor ligands (e. g., pyridine) as coupling partners, the latter not being able to poison the Cu‐catalyst by a possible and irreversible coordination to its metallic center.

The proper combination of either EG or 1,3‐propanediol (PD) with ChCl furnished reactive eutectic mixtures (ChCl/EG 1 : 2 mol mol^−1^, ChCl/PD 1 : 2 mol mol^−1^) enabling efficient C−O forming processes with brominated or iodinated (hetero)arenes **1** **a**,**d**,**g**,**h**, ending up with the isolation of products **2** **e**–**h** and **2** **j**–**l** in 75–90 % yield. Also, the use of a chlorinated electron‐deficient derivative like **1** **i** provided the expected adduct **2** **i** in 70 % yield, although at longer reaction time (24 h) and higher temperature (100 °C). In the absence of a copper catalyst, however, **1** **i** neither reacted with ChCl/Gly nor with ChCl/EG, even at 100 °C after 24 h. A eutectic mixture containing an hydroxy acid like ChCl/L‐lactic acid (LA) (1 : 2 mol mol^−1^) proved to be a competent coupling partner as well, smoothly delivering in the reaction with **1** **a**,**d** the desired products **2** **m**,**n** in 60–73 % yield (Scheme 3).

Despite the recommendations of institutions and organizations like the Green Chemistry Institute Pharmaceutical Roundtable (GCIPR), the European Union, and the United States Environmental Protection Agency (EPA) about the need to replace conventional hazardous VOCs with safer and bio‐based media,^[19]^ there are still very few examples of the employment of DESs in the synthesis of active pharmaceutical ingredients (APIs).[^13d^, ^13f^, ^13j^, ^14j^, ^15b^, ^20^] Aryloxypropanediols are prevalent motifs in pharmaceutically relevant compounds such as Guaiphenesin (**2** **o**),^[21]^ an expectorant drug useful in patients with stable chronic bronchitis, Mephenesin (Tolseron) (**2** **p**),^[22]^ used to treat muscle spasticity in Parkinson's disease and multiple sclerosis, and Chlorphenesin (**2** **q**),^[23]^ used as muscle relaxant and as antifungal agent and biocide in cosmetics (Scheme 3).

These drugs are usually prepared by nucleophilic attack of the suitable phenols on glycidol, epichlorohydrin, or 1‐chloroglycerol, which are toxic agents.^[24]^ The functionalization of glycerol with phenols, through in‐situ formation of glycerol carbonate, represents an interesting, less impactful alternative.^[25]^ Working at 105–110 °C with excess Gly (3 equiv.) and with reaction time up to 28 h, however, two competitive reaction/carbonatation pathways always took place, leading to the formation of two side products (2‐aryloxy‐1,3‐propanediols and 1,3‐diaryloxypropan‐2‐ols) in 4–8 % yield.

Addition of commercially available aryl halides **1** **j**–**l** to the eutectic mixture ChCl/Gly under open‐flask conditions provided with high chemo‐ and regioselectivity the desired target compounds **2** **o**–**q** with remarkable yields (70–98 %) (Scheme 3). The separation of regioisomers (formed in up to 20 % yield) was achieved by recrystallization from MeOH (see the Experimental Section). The robustness of these transformations in the above DES was ascertained during a scale‐up study. Performing the synthesis on a 2 g scale under the best conditions (10 g ChCl/Gly, 5 mol% CuI, 1 equiv. K_2_CO_3_, 80 °C in air) afforded products **2** **o**–**q** in similar yields (70–96 %, 1.54–2.01 g) after 6 h reaction time (Scheme 3).

**Figure 1:**
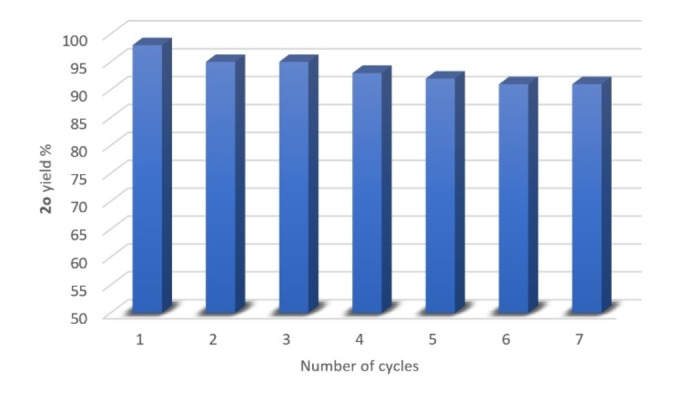
Recycling of CuI, DES, and base in the coupling reaction between 1‐iodo‐2‐methoxybenzene (**1** **j**) and Gly from ChCl/Gly in the synthesis of Guaiphenesin (**2** **o**). Yields were determined by ^1^H NMR spectroscopy using CH_2_Br_2_ as the internal standard.

Recycling studies were then conducted. The coupling of 1‐iodo‐2‐methoxybenzene (**1** **j**, 1.0 g) with Gly of the corresponding ChCl‐based eutectic mixture (5.0 g) was chosen as a model reaction as it provided Guaiphenesin (**2** **o**) in almost quantitative yield (98 %). Once the stirring was stopped after 6 h reaction time, extraction with cyclopentyl methyl ether (1 mL), an environmentally responsible solvent,^[26]^ afforded **2** **o** in 98 % yield (^1^H NMR spectroscopy) (Figure 1, number of cycles=1), but leaving the copper catalyst and the base in the eutectic mixture (see the Supporting Information). Then, after addition of fresh substrate, the catalyst, DES, and base could successfully be re‐used for further reaction runs. As shown in Figure 1, the catalyst remained active for over 7 cycles, with a decrease in the final yield of **2** **o** of up to 4 %: 95 % (second run), 95 % (third run), 93 % (fourth run), 92 % (fifth run), 91 % (sixth run), and 91 % (seventh run). There was no need to add Gly after each cycle because the resulting eutectic mixture (ChCl/Gly 1 : 1 mol mol^−1^; see the Supporting Information) proved to be still effective in promoting the etherification reaction (Table 1, entry 14). There was no need either to add additional K_2_CO_3_ after each cycle as 1 equiv. base was enough for 7 cycles. Because the medium employed (ChCl/Gly) is basic (pH=7.50),^[27]^ it may plausibly be playing an active role in regenerating the base (Scheme 2). Recent studies have also unveiled some critical roles played by base in the Ullmann‐type coupling catalyzed by Cu^I^ in the presence of neutral and bidentate ancillary ligands, like those of assisting other steps of the catalytic cycle, thereby accelerating the cross‐coupling reaction.^[28]^ After seven cycles, the isolation of **2** **o** provided 7.08 g of product with an E‐factor value (kg waste per kg product)^[29]^ of only 5.76 (see the Supporting Information).

## Conclusions

We have developed a sustainable and selective Cu‐catalyzed approach for the Ullmann‐type aryl alkyl ether synthesis by reacting various (hetero)aryl halides with alcohols, which are active components of biodegradable choline chloride‐based eutectic mixtures. These reactions proceed with a good substrate scope in air within 6 h at 80 °C (100 °C in the case of aryl chlorides), with K_2_CO_3_ as the base, Cu^I^ or Cu^II^ species as catalysts (up to 5 mol%), and in the absence of additional ligands, thereby offering an efficient pathway to various functionalized aryloxy derivatives with high regio‐ and chemoselectivity in up to 98 % yield. Of note, this simple protocol allows the valorization of naturally occurring polyols into high added‐value products (pharmaceutical drugs). Moreover, an effective recycling of the deep eutectic solvent (DES), base, and catalyst for up to 7 cycles with an E‐factor as low as 5.76 is presented. The methodology was successfully applied for the synthesis of pharmaceutically relevant aryloxypropanediols like Guaiphenesin, Mephenesin, and Chlorphenesin on a 2 g scale. This reinforces the argument that DESs can act as useful bio‐based solvents/reagents/ligands/catalysts for carrying out an environmentally friendly and controllable synthesis of active pharmaceutical ingredients.

## Experimental Section

**General procedure for synthesis of target compounds 2** **o–q on a 2** **g scale**: CuI (5 mol%, 0.4 mmol, 80.7 mg), 1‐iodo‐2‐methoxybenzene (**1** **j**, 8.5 mmol, 2 g), 1‐bromo‐2‐methylbenzene (**1** **k**, 11.7 mmol, 2 g) or 1‐chloro‐4‐iodo‐benzene (**1** **l**, 8.4 mmol, 2 g), and the base (K_2_CO_3_, 1 equiv.) were suspended in 10 g DES (ChCl/Gly, 1 : 2 mol mol^−1^), under air, in a vial with a Teflon screw tap. The corresponding mixture was vigorously stirred at 80 °C and monitored by thin‐layer chromatography. After 6 h, the mixture was cooled to room temperature and 10 mL of H_2_O was added. Then, the mixture was extracted with cyclopentyl methyl ether (3×10 mL), and the organic phase was dried over anhydrous Na_2_SO_4_ and filtered over a celite pad. In the case of **2** **o**, after evaporation of the solvent under reduced pressure, the crude was purified by crystallization from MeOH to provide the desired product in 98 % yield (1.65 g). For the synthesis of **2** **p** and **2** **q**, after evaporation of the solvent under reduced pressure, the crude was purified by flash‐chromatography on silica gel (CH_2_Cl_2_/MeOH 9 : 1), followed by recrystallization from MeOH, to provide **2** **p** in 75 % yield (1.60 g) and **2** **q** in 70 % yield (1.18 g).

## Conflict of interest

The authors declare no conflict of interest.

## Supporting information

As a service to our authors and readers, this journal provides supporting information supplied by the authors. Such materials are peer reviewed and may be re‐organized for online delivery, but are not copy‐edited or typeset. Technical support issues arising from supporting information (other than missing files) should be addressed to the authors.

Supporting Information
