# Supplementary material for: Ligand‐Free Copper‐Catalyzed Ullmann‐Type C−O Bond Formation in Non‐Innocent Deep Eutectic Solvents under Aerobic Conditions
Source: ChemSusChem. 2021 Dec 6;15(1):e202102211. doi: 10.1002/cssc.202102211 (PMC9299726; doi:10.1002/cssc.202102211)

ChemSusChem

Supporting Information

# Ligand-Free Copper-Catalyzed Ullmann-Type C—O Bond Formation in Non-Innocent Deep Eutectic Solvents under Aerobic Conditions

Andrea Francesca Quivelli, Manuela Marinò, Paola Vitale, Joaquín García-Álvarez,* Filippo M. Perna,* and Vito Capriati*© 2021 The Authors. ChemSusChem published by Wiley-VCH GmbH. This is an open access article under the terms of the Creative Commons Attribution License, which permits use, distribution and reproduction in any medium, provided the original work is properly cited.

## Author Contributions

A.Q. Conceptualization:Equal; Data curation:Equal; Formal analysis:Lead; Investigation:Lead; Methodology:Equal; Validation:Equal; Writing – original draft:Equal; Writing – review & editing:Equal
M.M. Data curation:Supporting; Formal analysis:Supporting; Investigation:Supporting; Writing – review & editing:-Supporting
P.V. Conceptualization:Equal; Formal analysis:Equal; Investigation:Equal; Methodology:Equal; Validation:Equal; Writing – review & editing:Equal
J.G.-A. Conceptualization:Equal; Funding acquisition:Equal; Investigation:Equal; Methodology:Equal; Supervision:Equal; Writing – review & editing:Equal
F.P. Conceptualization:Equal; Data curation:Lead; Formal analysis:Equal; Investigation:Equal; Methodology:Equal; Supervision:Equal; Writing – original draft:Equal; Writing – review & editing:Equal
V.C. Conceptualization:Equal; Funding acquisition:Lead; Methodology:Equal; Project administration:Lead; Supervision:Equal; Validation:Equal; Writing – original draft:Equal; Writing – review & editing:Equal

# Supporting Information

## Ligand-free Copper-Catalyzed Ullmann-type C–O Bond Formation in Non-innocent Deep Eutectic Solvents under Aerobic Conditions

Andrea Francesca Quivelli,[a] Manuela Marinò,[a] Paola Vitale,[a] Joaquín García-Álvarez,*[,b]
Filippo Maria Perna*[,a] and Vito Capriati*[,a]

[a] Università di Bari "Aldo Moro", Consorzio C.I.N.M.P.I.S. Via E. Orabona 4, I-70125, Bari, Italy
[b] Laboratorio de Química Sintética Sostenible (QuimSinSos), Departamento de Química Orgánica e Inorgánica (IUQOEM), Centro de Innovación en Química Avanzada (ORFEO-CINQA), Universidad de Oviedo, Spain

## Table of Contents

1. General Methods S2
2. Experimental Procedures S3
   2.1 Ullmann C-O coupling reaction between bromobenzene (**1a**) and Gly to give adducts **2a** and **3a**. Typical procedure. S3
   2.2 Synthesis of target compounds **2o–q** on a 2-gram scale. Typical procedure. S3
   2.3 Recycling of Cu catalyst, DES and base in the coupling reaction of 1-iodo-2-methoxybenzene (**1j**) to prepare Guaiphenesin (**2o**). General procedure. S4
   2.4 E Factor determination for the synthesis of Guaiphenesin (**2o**). S5
   2.5 Optimization of the Ullmann C–O coupling reaction between aryl halides and alcohols in DESs. S6
   2.6 Kinetics profile of the reaction between bromobenzene (**1a**) and Gly to give adduct **2a** S7
3. Spectroscopic data S8
4. $^{1}H$ and $^{13}C$ NMR spectra S13

## 1. General Methods

Deep Eutectic Solvents [choline chloride (ChCl)/glycerol (Gly) (1:2 mol mol$^{-1}$); ChCl/Gly (1:1 mol mol$^{-1}$); ChCl/Gly (1:3 mol mol$^{-1}$); L-Pro/Gly (2:5 mol mol$^{-1}$); betaine/Gly (1:2 mol mol$^{-1}$); ChCl/ethylene glycole (1:2 mol mol$^{-1}$); ChCl/1,3-propanediol (1:2 mol mol$^{-1}$); ChCl/L-lactic acid (1:2 mol mol$^{-1}$); acetic acid /menthol (1:1 mol mol$^{-1}$); decanoic acid/menthol (1:2 mol mol$^{-1}$) were prepared by heating under stirring at 60–80 °C for 10–30 min the corresponding individual components until a clear solution was obtained. For $^{1}$H NMR (600 MHz) and $^{13}$C NMR (150 MHz), $CDCl_3$ was used as the solvent; chemical shifts are reported in parts per million (δ). FT-IR spectra were recorded on a Perkin-Elmer 681 spectrometer. GC analyses were performed on a HP 6890 model, Series II by using a HP1 column (methyl siloxane; 30 m × 0.32 mm × 0.25 µm film thickness). Analytical thin-layer chromatography (TLC) was carried out on pre-coated 0.25 mm thick plates of Kieselgel 60 $F_{254}$; visualization was accomplished by UV light (254 nm) or by spraying a solution of 5 % (w/v) ammonium molybdate and 0.2 % (w/v) cerium(III) sulfate in 100 mL 17.6 % (w/v) aq. sulphuric acid and heating to 473 K until blue spots appeared. Chromatography was run by using silica gel 60 with a particle size distribution 40–63 µm and 230–400 ASTM. GC-MS analyses were performed on a HP 5995C model. Cyclopentyl methyl ether (CPME) was used as the solvent in the work-up procedures. High-resolution mass spectrometry (HRMS) analyses were performed using a Bruker microTOF QII mass spectrometer equipped with an electrospray ion source (ESI). CPME was provided by Zeon Europe GmbH. Other reagents and solvents, unless otherwise specified, were purchased from Sigma-Aldrich (Sigma-Aldrich, St. Louis, MO, USA) and were used without any further purification. Full characterization data, including copies of $^{1}$H and $^{13}$C NMR spectra, have been reported for both the newly synthesized compounds and the known compounds. The following abbreviations have been used to explain the multiplicities: s = singlet, d = doublet, t = triplet, q = quartet, m = multiplet, quin = quintuplet, br = broad, dd = double doublet, dt = double triplet.

## 2. Experimental Procedures

### 2.1 Ullmann C-O coupling reaction between bromobenzene (1a) and Gly to give adducts 2a and 3a. Typical procedure.

CuI (5 mol%, 0.05 mmol, 9.5 mg), bromobenzene (**1a**, 1 equiv, 1 mmol, 79 mg, 53 µL), and the base ($K_2CO_3$, 1 equiv, 1 mmol, 138 mg) were suspended in 1 g DES (ChCl/Gly, 1:2 mol $mol^{-1}$), under air, in a vial with a Teflon screw tap, The corresponding mixture was vigorously stirred at 80 °C, and monitored by TLC. After 6 h, the mixture was cooled to room temperature and 1 mL of $H_2O$ was added. Then, the mixture was extracted with CPME (3 × 1 mL), the organic phase was dried over anhydrous $Na_2SO_4$, and filtered over a celite pad. Evaporation of the solvent under reduced pressure afforded the crude that was purified by flash-chromatography on silica gel ($CH_2Cl_2$/MeOH 9:1) to provide the desired adduct **2a** as a mixture with **3a** (164 mg, ratio **2a**:**3a**: 4:1; **2a** yield: 77%, by $^1$H NMR).

### 2.2 Synthesis of target compounds 2o–q on a 2-gram scale. Typical procedure.

CuI (5 mol%, 0.4 mmol, 80.7 mg), 1-iodo-2-methoxybenzene (**1j,** 8.5 mmol, 2 g) or 1-bromo-2-methylbenzene (**1k,** 11.7 mmol, 2 g) or 1-chloro-4-iodo-benzene (**1l,** 8.4 mmol, 2 g), and the base ($K_2CO_3$, 1 equiv) were suspended in 10 g DES (ChCl/Gly, 1:2 mol $mol^{-1}$), under air, in a vial with a Teflon screw tap. The corresponding mixture was vigorously stirred at 80 °C, and monitored by TLC. After 6 h, the mixture was cooled to room temperature and 10 mL of $H_2O$ was added. Then, the mixture was extracted with CPME (3 × 10 mL), the organic phase was dried over anhydrous $Na_2SO_4$, and filtered over a celite pad. In the case of **2o**, after evaporation of the solvent under reduced pressure, the crude was purified by crystallization from MeOH to provide the desired product in 98 % yield (1.65 g). As for the synthesis of **2p** and **2q**, after evaporation of the solvent under reduced pressure, the crude was purified by flash-chromatography on silica gel ($CH_2Cl_2$/MeOH 9:1), followed by recrystallization from MeOH, to provide **2p** in 75% yield (1.60 g) and **2q** in 70% yield (1.18 g).

**2.3 Recycling of Cu catalyst, DES and base in the coupling reaction of 1-iodo-2-methoxybenzene (1j) to prepare Guaiphenesin (2o). General procedure.**

CuI (5 mol%, 0.2 mmol, 40.8 mg), 1-iodo-2-methoxybenzene (**1j**, 4.3 mmol, 1.0 g), and the base ($K_2CO_3$, 1 equiv, 5.4 mmol, 759 mg) were sequentially added to 12 g DES (ChCl/Gly, 1:2 mol mol$^{-1}$), under air, in a vial with a Teflon screw tap. The reaction mixture was vigorously stirred at 80°C for 6 h (monitoring the complete consumption of the starting material by TLC), and then cooled to room temperature. The product was extracted with CPME (5 mL), leaving the catalyst and the base in the eutectic mixture, which were re-used for further reaction runs. The organic layer was filtered through a celite pad, the volatile was evaporated under vacuum, and the crude so obtained was analyzed by $^1$H NMR to determine the yield of **2o** ($CH_2Br_2$ was used as the internal standard). New, fresh reagents were then added to the recovered eutectic mixture, and the whole procedure was repeated for seven times without any significant loss of the catalyst activity (91% yield after the seven cycle). After seven cycles, Guaiphenesin (**2o**) was isolated in 7.08 g (see Section 2.4).

**Determination of the amount of Gly from ChCl/Gly to form the product 2o in recycling procedure**. During each cycle, 4.3 mmol of Gly reacted in cross-coupling reaction per 1 g substrate (**1j**) *en route* to **2o**. After 7 cycles, 30.1 mmol of Gly was consumed, and the composition of DES (ChCl/Gly) changed from 1:2 mol mol$^{-1}$ (12 g DES includes 37.4 mmol ChCl and 74.3 mmol Gly) to about 1:1 mol mol$^{-1}$ (37.4 mmol ChCl and 37.4 mmol Gly).

## 2.4 E Factor determination for the synthesis of Guaiphenesin (2o).

According to its original definition (*Green Chem*. **2007**, *9*, 1273), the Sheldon E factor value (total mass of waste/mass of product) takes into account only the mass of waste generated in a process, and its calculation is performed by simply dividing the sum of the molecular weight of all substances produced by molecular weight of the desired products, with reference to the stoichiometric equation. Thus, the amount of silica gel, the celite pad, the drying agents, and the mass of eluent solvent used for chromatography are usually not included in the calculation. We have followed this general equation in our own calculation.

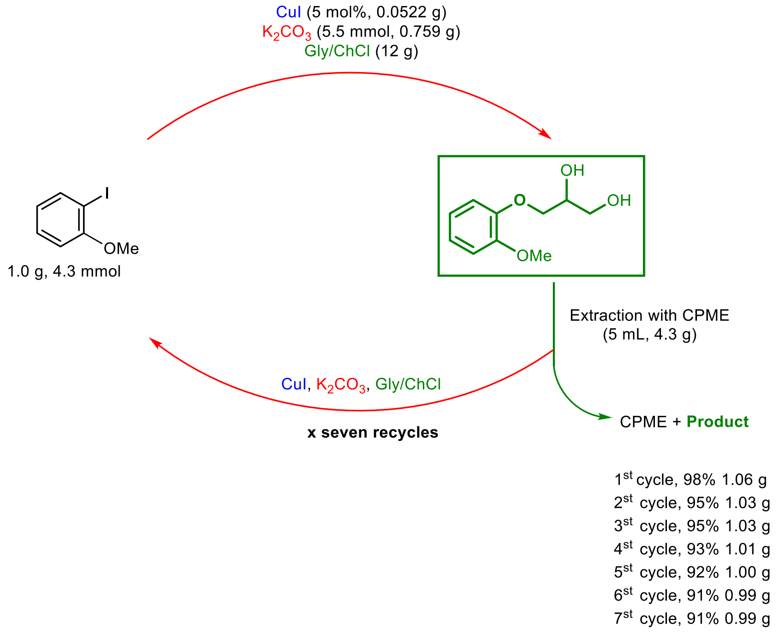


Note: CPME: 0.86 g/mL, at 25 °C.

*Total amount of reactants*: 12 g + 7×1 g + 0.052 g + 0.759 g + 7×4.3 g = 49.9 g

*Amount of final product*: 7.38 g

*Amount of waste*: 49.9 g – 7.38 g = 42.52 g

*E-Factor* = amount of waste/amount of product = 42.52 g/7.38 g = **5.76.**

## 2.5 Optimization of the Ullmann C-O coupling reaction between aryl halides and alcohols in DESs.[a]

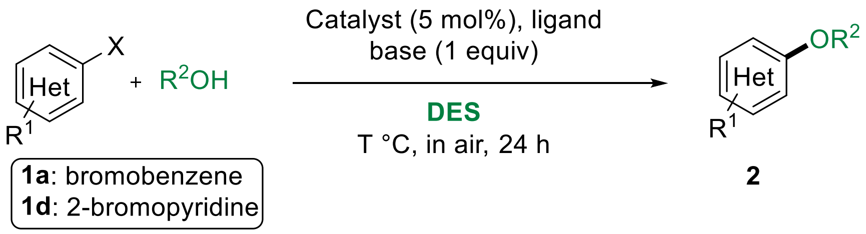


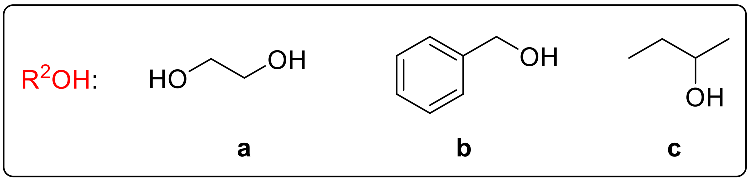


| Entry | **1** | $R^2OH$ | DES | Base | Cat. | Ligand | T °C | **2** yield |
|---|---|---|---|---|---|---|---|---|
| 1 | **1a** | **a** | ChCl/urea | $K_2CO_3$ | CuI[b] | / | 130 | / |
| 2 | **1a** | **a** | ChCl/urea | $K_2CO_3$ | $CuCl_2$[b] | / | 130 | / |
| 3 | **1a** | **a** | ChCl/urea | $K_2CO_3$ | CuO | / | 130 | / |
| 4 | **1a** | **a** | ChCl/urea | $K_2CO_3$ | $Pd(OAc)_2$[b] | / | 130 | / |
| 5 | **1a** | **b** | ChCl/urea | $K_2CO_3$ | $CuCl_2$ | 1,10-Phen[c] | 110 | / |
| 6 | **1a** | **b** | ChCl/urea | $Cs_2CO_3$ | CuI | 1,10-Phen[c] | 110 | / |
| 7 | **1a** | **b** | ChCl/urea | $K_2CO_3$ | $CuCl_2$ | L-proline[c] | 110 | / |
| 8 | **1a** | **b** | ChCl/urea | $K_2CO_3$ | $CuCl_2$ | *N,N*-dimethylglycine[c] | 110 | / |
| 9 | **1a** | **b** | ChCl/urea | $K_2CO_3$ | $CuCl_2$ | ethylenediamine[c] | 110 | / |
| 10 | **1d** | **a** | ChCl/urea | $K_2CO_3$ | $CuCl_2$ | / | 130 | / |
| 11 | **1d** | **a** | ChCl/urea | $K_2CO_3$ | $CuCl_2$ | / | 70 | / |
| 12 | **1d** | **c** | ChCl/urea | *t*-BuOK | $CuCl_2$ | / | 70 | / |
| 13 | **1d** | **b** | ChCl/urea | $CH_3CO_2Na$ | $CuCl_2$ | / | 70 | / |
| 14 | **1d** | **b** | acetic acid/menthol | $K_2CO_3$ | $CuCl_2$ | / | 100 | / |
| 15 | **1d** | **a** | decanoic acid/menthol | $K_2CO_3$ | $CuCl_2$ | / | 100 | / |
| 16 | **1d** | **/** | ChCl/D-fructose | $K_2CO_3$ | $CuCl_2$ | / | 100 | [d] |

[a] Reaction conditions: 1.0 g DES per 1.0 mmol of **1** and 1 mmol of $R_2OH$. DES: choline chloride (ChCl)/Urea (1:2 mol $mol^{-1}$), acetic acid /menthol (1:1 mol $mol^{-1}$), decanoic acid/menthol (1:2 mol $mol^{-1}$), ChCl/D-fructose (1:2 mol $mol^{-1}$). [b] Reaction also performed with 20 mol% of catalyst and of 48 h reaction time. [c] Ligand: 20 mol%. [d] Complex mixture of regioisomeric products.

**2.6 Kinetics profile of the reaction between bromobenzene (1a) and Gly to give adduct 2a.**

The reaction is characterized by a slow kinetics delivering adduct **2a** in a lower yield at shorter reaction time than 6 h.

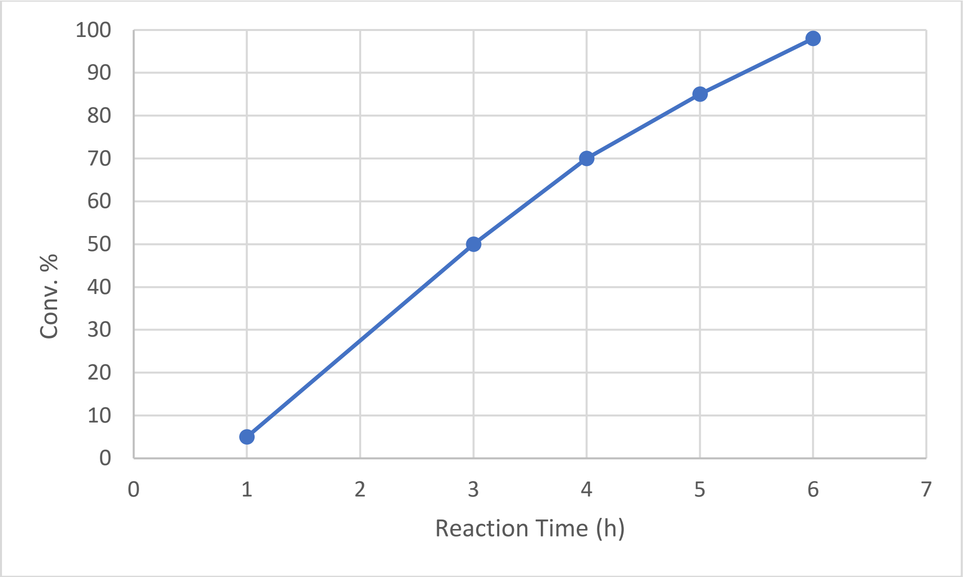


**Figure S1.** Conversion vs. time profile for the synthesis of **2a** at 80 °C, in air. Yields were determined by $^1$H NMR using $CH_2Br_2$ as the internal standard.

**3. Spectroscopic data**

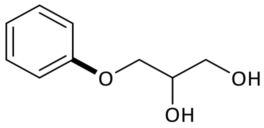

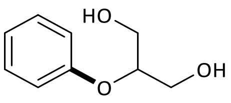

**3-Phenoxypropane-1,2-diol (2a), 2-phenoxypropane-1,3-diol (3a):** inseparable mixture of regioisomers: 98% yield, **2a**:**3a** ratio: 4:1, yellow oil. $^{1}$H NMR (600 MHz, $CDCl_3$) δ 1.74 (br s, 2 H **2a** + 2 H **3a**), 3.76 (dd, *J* = 11.2, 5.7 Hz, 1 H **2a**), 3.85 (dd, *J* = 11.2, 3.7 Hz, 1 H **2a**), 3.93 (d, *J* = 5.4 Hz, 4 H **3a**), 3.98–4.05 (m, 2 H **2a**), 4.07–4.16 (m, 1 H **2a**), 4.45 (quin, *J* = 5.4 Hz, 1 H **3a**), 6.90–7.01 (m, 3 H **2a** + 3 H **3a**) 7.28–7.32 (m, 2 H **2a** + 2 H **3a**); $^{13}$C NMR (150 MHz, $CDCl_3$) δ 62.2 (**3a**), 63.7 (**2a**), 69.1 (**2a**), 70.3 (**2a**), 78.4 (**3a**),114.5 (**2a**), 116.3 (**3a**), 121.3 (**2a**), 121.8 (**3a**), 129.6 (**2a**), 129.7 (**3a**), 157.6 (**3a**), 158.4 (**2a**); FT-IR (film, cm$^{-1}$): 3350, 2920, 2850, 1599, 1494, 1243, 1041, 752; GC/MS (70 eV) *m/z* (%) **2a:** 168 (M$^{+}$, 68), 137 (62), 119 (25), 108 (20), 103 (17), 94 (100), 77 (62), 65 (25); **3a**: 168 (M$^{+}$, 65), 137 (68), 119 (27), 108 (24), 103 (13), 94 (100), 77 (55). HRMS (ESI) *m/z* calcd for $[C_9H_{12}O_3 + Na]^+$: 191.0668; found: 191.0669.

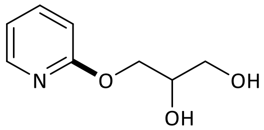

**3-(Pyridin-2-yloxy)propane-1,2-diol (2b)**: 86% yield, yellow oil. $^{1}$H NMR (600 MHz, $CDCl_3$) δ 2.12 (br s, 1 H), 3.31 (br s, 1 H), 3.62–3.73 (m, 2 H), 3.99–4.05 (m, 1 H), 4.43–4.44 (m, 2 H), 6.79 (d, *J* = 8.3 Hz, 1 H), 6.90 (t, *J* =6 Hz, 1 H), 7.60 (t, *J* = 6 Hz, 1 H), 8.08 (d, *J* = 6 Hz, 1 H); $^{13}$C NMR (150 MHz, $CDCl_3$) δ 63.3, 68.2, 71.0, 110.5, 116.6, 139.4, 146.3, 163.8; FT-IR (film, cm$^{-1}$): 3350, 2939, 1679, 1598, 1573, 1471, 1434, 1287, 1120, 1047, 779; GC/MS (70 eV) *m/z* (%): 169 (M$^{+}$, 8), 138 (100), 120 (11), 109 (15), 108 (32), 96 (78), 95 (36), 80 (12), 79 (24), 78 (43), 67 (37). HRMS (ESI) *m/z* calcd for $[C_8H_{11}NO_3 + Na]^+$: 192.0637; found: 192.0630.

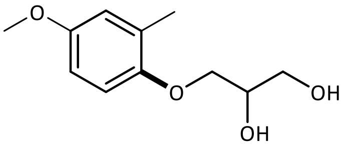

**3-(4-Methoxy-2-methylphenoxy)propane-1,2-diol (2c):** 80% yield, yellow oil, $^{1}$H NMR (600 MHz, $CDCl_3$) δ 2.22 (s, 3 H), 2.41 (br s, 2 H), 3.76 (s, 3 H), 3.82–3.89 (m, 2 H), 3.92–4.00 (m, 2 H), 4.08–4.15 (m, 1 H), 6.63–6.68 (m, 1 H), 6.72 (s, 1 H), 6.75 (d, *J* = 6 Hz, 1 H); $^{13}$C NMR (150 MHz, $CDCl_3$) δ 55.6, 63.8, 70.3, 70.6, 110.7, 112.8, 117.0, 128.1, 151.3, 153.5; FT-IR (film, cm$^{-1}$): 3150, 2959, 1676, 1580, 1556, 1461, 1334, 1287, 1121, 1047, 600; GC/MS (70 eV) *m/z* (%): 212 (M$^{+}$, 30), 138 (100), 123 (11), 109 (15), 101 (32), 91 (18), 77 (24), 67 (37), 57 (15), 41 (11). HRMS (ESI) *m/z* calcd for $[C_{11}H_{16}O_6 + H]^+$: 213.1121; found: 213.1120.

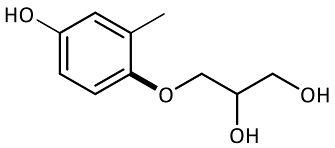


**3-(4-Hydroxy-2-methylphenoxy)propane-1,2-diol (2d):** 90% yield, yellow oil. $^1$H NMR (500 MHz, $CDCl_3$) δ $^1$H NMR (300 MHz, $CDCl_3$) δ 2.08 (s, 3 H), 3.69–3.72 (m, 1 H), 3.74–3.82 (m, 1 H), 3.96–4.02 (m, 2 H), 4.12–4.18 (m, 1 H), 6.64–6.66 (m, 1 H), 6.67 (s, 1 H), 7.13 (d, *J* = 9.0 Hz, 1 H); $^{13}$C NMR (150 MHz, $CDCl_3$) δ 16.3, 63.9, 67.0, 70.5, 112.3, 112.8, 118.0, 121.0, 154.9, 156.5; FT-IR (film, cm$^{-1}$): 3250, 2929, 1779, 1589, 1573, 1571, 1424, 1290, 1120, 1047, 600; GC/MS (70 eV) *m/z* (%): 198 ($M^+$, 30), 166 (15), 149 (20), 137 (11), 124 (100), 107 (15), 95 (78), 77 (40), 67 (37), 55 (15), 43 (10). HRMS (ESI) *m/z* calcd for $[C_{10}H_{14}O_4 + H]^+$: 199.0965; found: 199.0966.

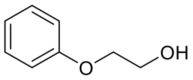


**2-Phenoxyetan-1-ol (2e):** 84% yield, colourless liquid. $^1$H NMR (600 MHz, $CDCl_3$) δ 3.98 (t, *J* = 6.0 Hz, 2 H), 4.10 (t, *J* = 6.0 Hz, 2 H), 6.93– 7.01 (m, 3 H), 7.24–7.26 (m, 2 H); $^{13}$C NMR (150 MHz, $CDCl_3$) δ 61.2, 69.2, 114.7, 121.1, 129.5, 159.9; FT-IR (film, cm$^{-1}$) 3388, 2911, 16455, 1211, 1032, 749, 701; GC/MS (70 eV) *m/z* (%): 138 ($M^+$, 6), 137 (22), 95 (9), 94 (100), 77 (32). HRMS (ESI) *m/z* calcd for $[C_8H_{10}O_2 + H]^+$: 139.0754; found: 139.0760.

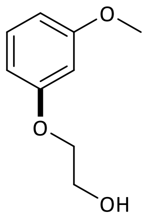


**2-(3-Methoxyphenoxy)ethan-1-ol (2f):** 75% yield, yellow oil. $^1$H NMR (600 MHz, $CDCl_3$) δ 2.10 (br s, 1 H), 3.78 (s, 3 H), 3.95 (t, *J* = 5.2 Hz, 2 H), 4.05 (t, *J* = 5.2 Hz, 2 H), 6.49 (t, *J* = 5.9 Hz 1 H), 6.49–6.53 (m, 2 H), 7.19 (t, *J* = 5.9 Hz, 1 H); $^{13}$C NMR (150 MHz, $CDCl_3$) δ 55.3, 61.4, 69.1, 101.0, 106.6, 106.7, 129.9, 159.8, 160.8; FT-IR (film, cm$^{-1}$): 3354, 2939, 2875, 1729, 1588, 1492, 1453, 1335, 1264, 1199, 1038, 994, 834, 761, 686; GC/MS (70 eV) *m/z* (%): 168 ($M^+$, 60), 125 (55), 124 (100), 107 (17), 96 (28), 95 (27), 94 (44), 81 (15), 77 (21). HRMS (ESI) *m/z* calcd for $[C_9H_{12}O_3 + Na]^+$: 191.0684; found: 191.0680.

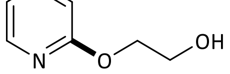


**2-(Pyridin-2-yloxy)ethan-1-ol (2g):** 80% yield, yellow oil. $^1$H NMR (600 MHz, $CDCl_3$): 3.91 (t, *J* = 6.0 Hz, 2 H), 4.22 (t, *J* = 6.0 Hz, 2 H), 6.75 (d, *J* = 12.0 Hz, 1 H), 6.87 (t, *J* = 6.0, 1 H), 7.56 (t, *J* = 6.0, 1 H), 8.10 (d, *J* = 6.0 Hz, 1 H); $^{13}$C NMR (150 MHz, $CDCl_3$): 62.1, 68.6, 110.9, 117.1, 139.0, 146.4, 162.3; FT-IR (film, cm$^{-1}$): 3363, 3017, 2942, 2878, 1663, 1596, 1570, 1475, 1432, 1312, 1287, 1047; GC-MS (70 eV) m/z (%): 139 ($M^+$, 3), 120 (25), 109 (43), 108 (41), 96 (88), 95 (100), 80 (25), 78 (91), 67 (84), 51 (26). HRMS (ESI) *m/z* calcd for $[C_7H_{10}NO_2 + H]^+$: 140.0706; found: 140.0705.

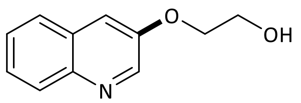

**2-(Quinolin-3-yloxy)ethan-1-ol (2h)**: 90% yield, yellow oil. $^{1}H$ NMR (600 MHz, $CDCl_3$) δ 4.07 (t, *J* = 6.0 Hz, 2 H), 4.21 (t, *J* = 6.0 Hz, 2 H), 7.38 (d, *J* = 2.8 Hz, 1 H), 7.48–7.52 (m, 1 H), 7.54–7.57 (m, 1 H), 7.71 (d, *J* = 8.2, 1 H), 8.04 (d, *J* = 8.2 Hz, 1 H), 8.67 (d, *J* = 2.8, Hz, 1 H); $^{13}C$ NMR (150 MHz, $CDCl_3$) δ 61.1, 69.7, 113.4, 126.8, 126.9, 127.2, 128.7, 128.9, 143.4, 144.5, 152.1. FT-IR (film, $cm^{-1}$): 3352, 3334, 3052, 2902, 2814, 2017, 1621, 1493, 1412, 1357, 1223, 1073; GC/MS (70 eV) *m/z* (%): 189 ($M^+$, 39), 146 (10), 145 (100), 128 (11), 90 (10), 89 (13). HRMS (ESI) *m/z* calcd for $[C_{11}H_{11}NO_2 + H]^+$: 190.0863; found: 190.0855.

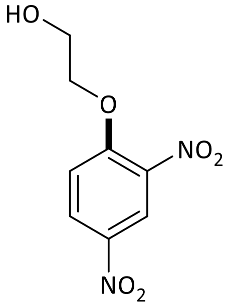

**2-(2,4-Dinitrophenoxy)ethan-1-ol (2i)**: 70% yield, yellow solid. $^{1}H$ NMR (600 MHz, $CDCl_3$) δ 3.75–3.78 (m, 4 H), 7.33 (d, *J* = 9.2 Hz, 1 H), 8.46 (d, *J* = 9.2 Hz, 1 H), 9.08 (s, 1 H); $^{13}C$ NMR (150 MHz, $CDCl_3$) δ 62.0, 70.6, 114.4, 122.0, 129.1, 140.7, 141.6, 157.6; FT-IR (film, $cm^{-1}$): 3344, 2935, 1729, 1581, 1493, 1345, 1265, 1198, 1038,, 995, 844, 762, 686; GC/MS (70 eV) *m/z* (%): 229 ($M^+$, 100), 228 (38), 227 (98), 185 (42), 183 (40), 158 (27), 155 (23), 149 (41), 91 (22), 89 (47), 77 (31). HRMS (ESI) *m/z* calcd for $[C_8H_8N_2O_6 + H]^+$: 229.0455; found: 229.0454.

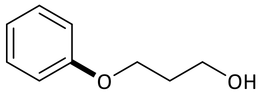

**3-Phenoxypropan-1-ol (2j):** 80% yield, colourless liquid. $^{1}H$ NMR (600 MHz, $CDCl_3$) δ 1.75 (s, 1 H), 2.05 (quin, *J* = 5.9 Hz, 2 H), 3.86–3.88 (m, 2 H), 4.13 (t, *J* = 5.9 Hz, 2 H), 6.98– 6.88 (m, 3 H), 7.24–7.32 (m, 2 H); $^{13}C$ NMR (150 MHz, $CDCl_3$) δ 31.9, 60.6, 65.7, 114.5, 120.9, 129.5, 158.7; FT-IR (film, $cm^{-1}$) 3392, 2917, 1640, 1032; GC/MS (70 eV) *m/z* (%): 153 ($M^+$, 2), 152 (25), 95 (9), 94 (100), 77 (8), 66 (8), 65 (8), 51 (3). HRMS (ESI) *m/z* calcd for $[C_9H_{12}O_2 + H]^+$: 153.0910; found: 153.0903.

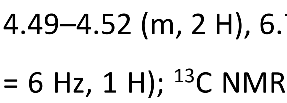

**3-(Pyridin-2-yloxy)propan-1-ol (2k)**: 75% yield, colourless liquid. $^{1}H$ NMR (600 MHz, $CDCl_3$) δ 1.97 (quin, 2 H), 3.34 (br s, 1 H), 3.70 (t, *J* = 5.8 Hz, 2 H), 4.49–4.52 (m, 2 H), 6.73 (d, *J* = 8.4 Hz, 1 H), 6.87 (t, *J* =8.4 Hz, 1 H), 7.57 (t, *J* = 8.4 Hz, 1 H), 8.11 (d, *J* = 6 Hz, 1 H); $^{13}C$ NMR (150 MHz, $CDCl_3$) δ 32.5, 58.8, 62.8, 111.3, 116.8, 138.9, 146.6, 164.1; FT-IR (film $cm^{-1}$): 3367, 3027, 2945, 2880, 1673, 1595, 1572, 1475, 1432, 1313, 1286, 1047; GC-MS (70 eV) m/z (%): 153 ($M^+$, 4), 123 (19), 122 (45), 108 (48), 96 (100), 95 (90), 79 (26), 78 (57), 67 (80), 51 (12). HRMS (ESI) m/z calcd for $[C_8H_{11}NO_2 + H]^+$: 154.0865; found: 154.0866.

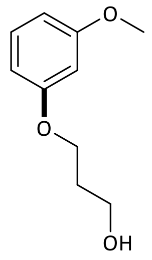


**3-(3-Methoxyphenoxy)propan-1-ol (2l)**: 75% yield, yellow oil. $^{1}$H NMR (600 MHz, $CDCl_3$) δ 1.66 (br s, 1 H), 1.99 (quin, 2 H), 3.74 (t, $J$ = 3.0 Hz, 2 H), 3.81 (s, 3 H), 4.11 (t, $J$ = 3.0 Hz, 2 H), 6.47 (s, 1 H), 6.48–6.52 (m, 2 H), 7.16 (t, $J$ = 6.0 Hz, 1 H); $^{13}$C NMR (150 MHz, $CDCl_3$) δ 31.4, 55.18, 58.88, 66.94, 101.00, 107.92, 109.39, 129.48, 159.71, 160.25; FT-IR (film, cm$^{-1}$): 3364, 2940, 2886, 1728, 1576, 1492, 1463, 1334, 1266, 1209, 1041, 984, 836, 760, 689; GC/MS (70 eV) $m/z$ (%): 182 ($M^+$, 70), 125 (55), 124 (100), 107 (17), 151 (10), 96 (28), 94 (44), 81 (15), 77 (21). HRMS (ESI) $m/z$ calcd for $[C_{10}H_{14}O_3 + H]^+$: 183.1016; found: 183.1015.

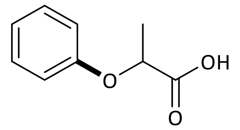


**2-Phenoxypropanoic acid (2m):** 73% yield, yellow oil. $^{1}$H NMR (600 MHz, $CDCl_3$) δ 1.45 (d, $J$ = 6.7 Hz, 3 H), 4.60 (q, $J$ = 7.0 Hz, 1 H), 6.82 (d, $J$ = 8.0 Hz, 2 H), 6.92 (t, $J$ = 7.4 Hz, 1 H), 7.19 (t, $J$ = 7.7 Hz, 2 H); $^{13}$C NMR (150 MHz, $CDCl_3$) δ 18.3, 73.3, 115.6, 121.7, 127.8, 157.2, 177.6; FT-IR (film, cm$^{-1}$): 3063, 2920, 2527, 1943, 1729, 1600, 1488, 1416, 1240, 1135, 753, 691; GC/MS (70 eV) $m/z$ (%): 165 (27), 121 (63), 94 (100), 85 (50), 77 (51), 71 (83), 57 (67), 43 (60). HRMS (ESI) $m/z$ calcd for $[C_9H_{10}O_3 - H]^-$: 165.0552; found: 165.0557.

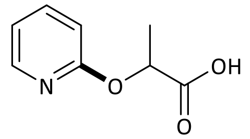


**2-(Pyridin-2-yloxy)propanoic acid (2n)**: 60% yield, yellow oil. $^{1}$H NMR (600 MHz, $CDCl_3$) δ 1.60 (d, $J$ = 5.3 Hz, 3 H), 5.26–5.28 (m, 1 H), 6.87 (d, $J$ = 7.6 Hz, 1 H), 6.94 (t, $J$ = 6.0, 1 H), 7.64 (t, $J$ = 6.0 Hz, 1 H), 8.09 (d, 1 H); $^{13}$C NMR (150 MHz, $CDCl_3$) δ 17.4, 81.4, 113.1, 119.1, 139.6, 146.1, 162.8, 174.9; FT-IR (film, cm$^{-1}$): 2921, 2850, 1732, 1597, 1573, 1471, 1434, 1273, 1250, 1132, 1097, 1047, 991, 946, 845, 778, 738; GC/MS (70 eV) $m/z$ (%): 166 (3), 123 (21), 122 (70), 108 (70), 96 (47), 95 (40), 79 (83), 78 (100), 67 (45), 51 (23), 43 (10). HRMS (ESI) $m/z$ calcd for $[C_8H_9NO_3 + Na]^+$: 190.0480; found: 189.0702.

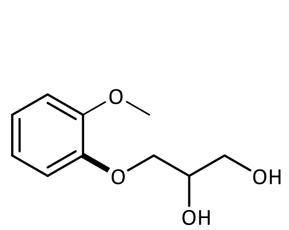


**3-(2-Methoxyphenoxy)propane-1,2-diol (2o)[1]:** 98% yield, white solid: mp 79–80 °C (mp 78–80 °C). $^{1}$H NMR (600 MHz, $CDCl_3$) δ 3.81–3.88 (m, 2 H), 3.89 (s, 3 H), 4.07–4.10 (m, 2 H), 4.17–4.20 (m, 1 H), 6.87–7.02 (m, 4 H); $^{13}$C NMR (150 MHz, $CDCl_3$) δ 55.8, 63.9, 70.0, 72.6, 112.2, 115.6, 121.1, 122.5, 148.2, 150.0; FT-IR (film, cm$^{-1}$): 3370, 2739, 1661, 1598, 1553, 1451, 1444, 1287, 1130, 1047, 501;GC/MS (70 eV) $m/z$ (%): 198 ($M^+$,

[1] M. A. Truscello, C. Gambarotti, M. Lauria, S. Auricchio, G. Leonardi, S. U. Shisodia, A. Citterio, *Green Chem.*, **2013**, *15*, 625.

55), 167 (15), 149 (20), 138 (11), 124 (100), 109 (70), 95 (20), 81 (40), 65 (20), 52 (15), 43 (10). HRMS (ESI) *m/z* calcd for $[C_{10}H_{14}O_4 + H]^+$: 199.0965; found: 199.0966.

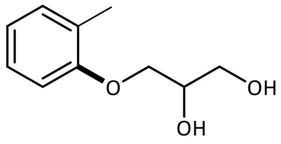

**3-(*ortho*-Tolyloxy)propane-1,2-diol (2p)**[1]: 75% yield, white solid, mp 66-67 °C. $^1H$ NMR (600 MHz, $CDCl_3$) δ 2.25 (s, 3 H), 3.78–3.82 (m, 1 H), 3.87–3.90 (m, 1 H), 4.07–4.09 (m, 2 H), 4.14–4.17 (m, 1 H), 6.84 (d, *J* = 6.0 Hz, 1 H), 6.91 (t, *J* = 6.0 Hz, 1 H), 7.16–7.18 (m, 2 H). $^{13}C$ NMR (150 MHz, $CDCl_3$) δ 15.7, 63.8, 68.7, 70.5, 111.3, 120.4, 126.9, 130.7, 156.5, 174.6; FT-IR (film, $cm^{-1}$): 3330, 2539, 1779, 1548, 1533, 1481, 1332, 1267, 1220, 1147, 770; GC/MS (70 eV) *m/z* (%): 182 ($M^+$, 30), 151 (15), 133 (10), 121 (20), 108 (100), 91 (78), 77 (43), 65 (37), 57 (10), 51 (6), 43 (6). HRMS (ESI) *m/z* calcd for $[C_{10}H_{14}O_4 + H]^+$: 183.1016; found: 183.1015.

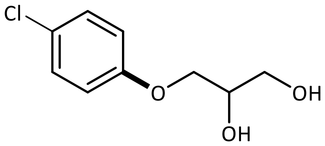

**3-(4-Chlorophenoxy)propane-1,2-diol (2q)**[1]**:** 70% yield, white solid: mp 74–75 °C. $^1H$ NMR (600 MHz, $CDCl_3$) δ 2.07 (br s, 2 H), 3.74–3.77 (m, 1 H), 3.83–3.86 (m, 1 H), 4.02–4.05 (m, 2 H), 4.10–4.13 (m, 1 H), 6.85 (d, *J* = 6.0 Hz, 2 H), 7.25 (d, *J* = 6.0 Hz, 2 H). $^{13}C$ NMR (150 MHz, $CDCl_3$) δ 63.5, 69.5, 70.3, 115.9, 126.3, 129.4, 157.0; FT-IR (film, $cm^{-1}$): 3352, 2940, 1679, 1588, 1554, 1461, 1438, 1277, 1121, 1047, 760; GC/MS (70 eV) *m/z* (%): 202 ($M^+$, 30), 153 (15), 141 (10), 128 (100), 111 (15), 99 (36), 93 (12), 75 (24), 65 (37), 50 (10), 43 (6). HRMS (ESI) *m/z* calcd for $[C_9H_{11}ClO_3 + H]^+$: 203.0469; found: 203.0468.

## 4. $^{1}H$ and $^{13}C$ NMR spectra

$^{1}H$ NMR, 600 MHz, $CDCl_3$

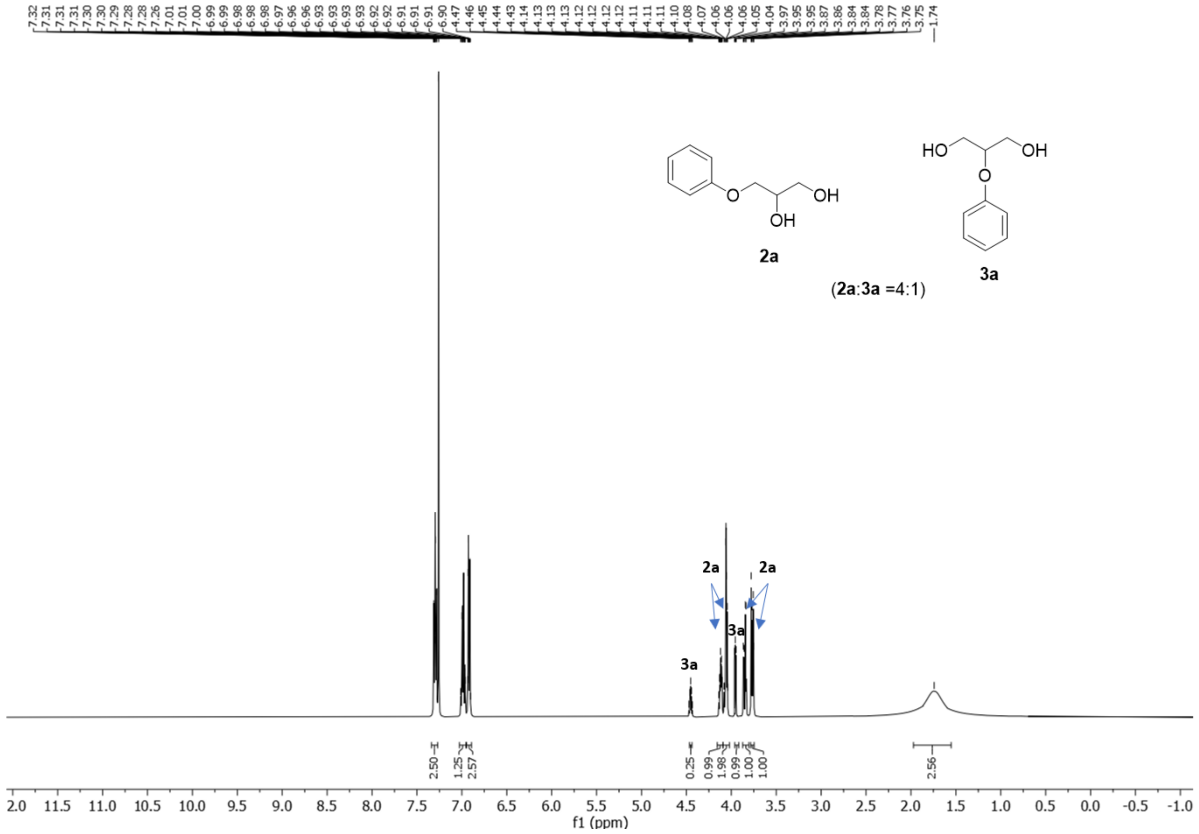


$^{13}C$ NMR, 150 MHz, $CDCl_3$

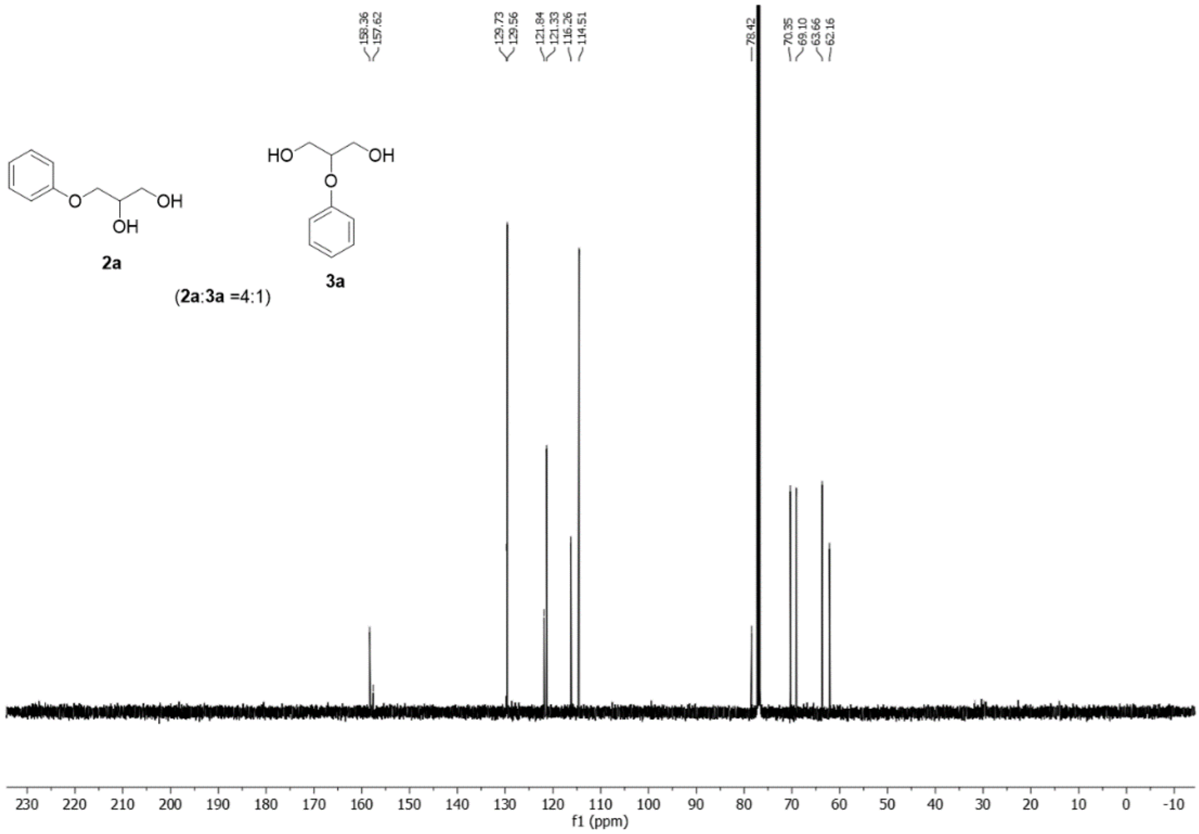

$^{1}$H NMR, 600 MHz, $CDCl_3$

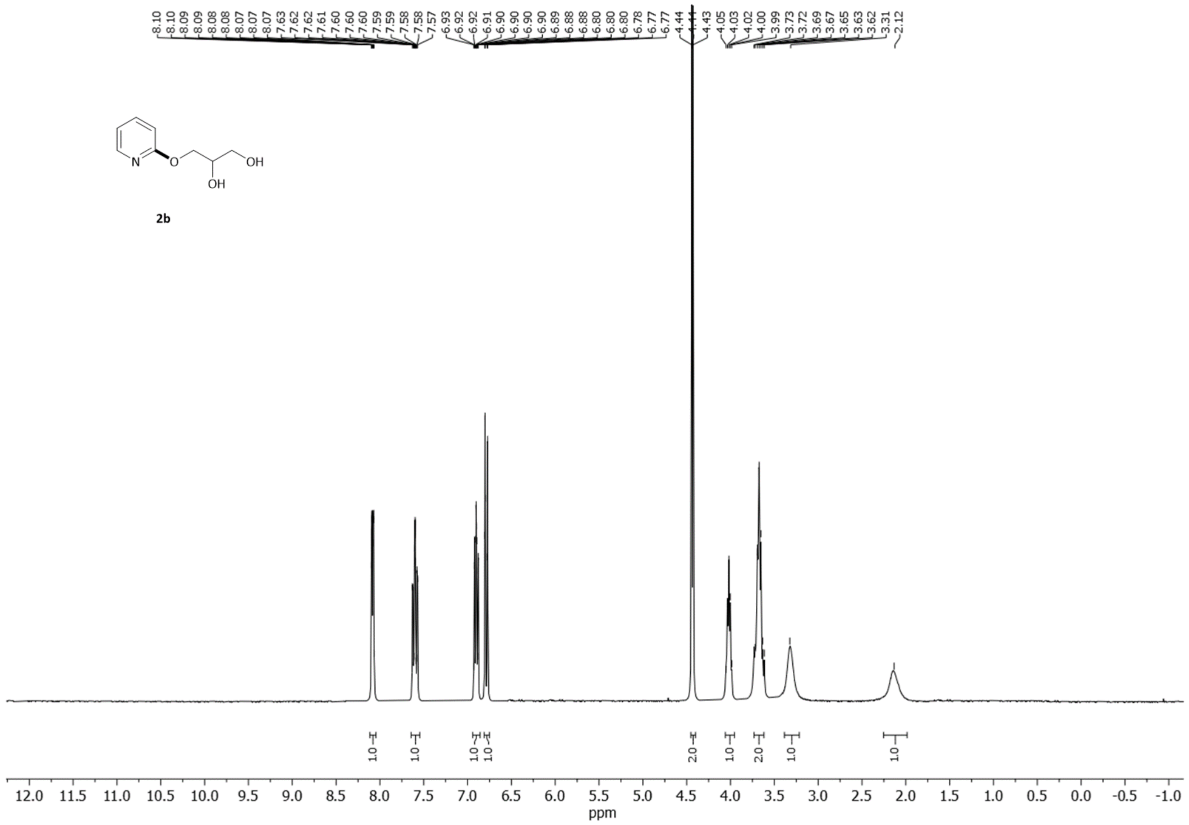

$^{13}$C NMR, 150 MHz, $CDCl_3$

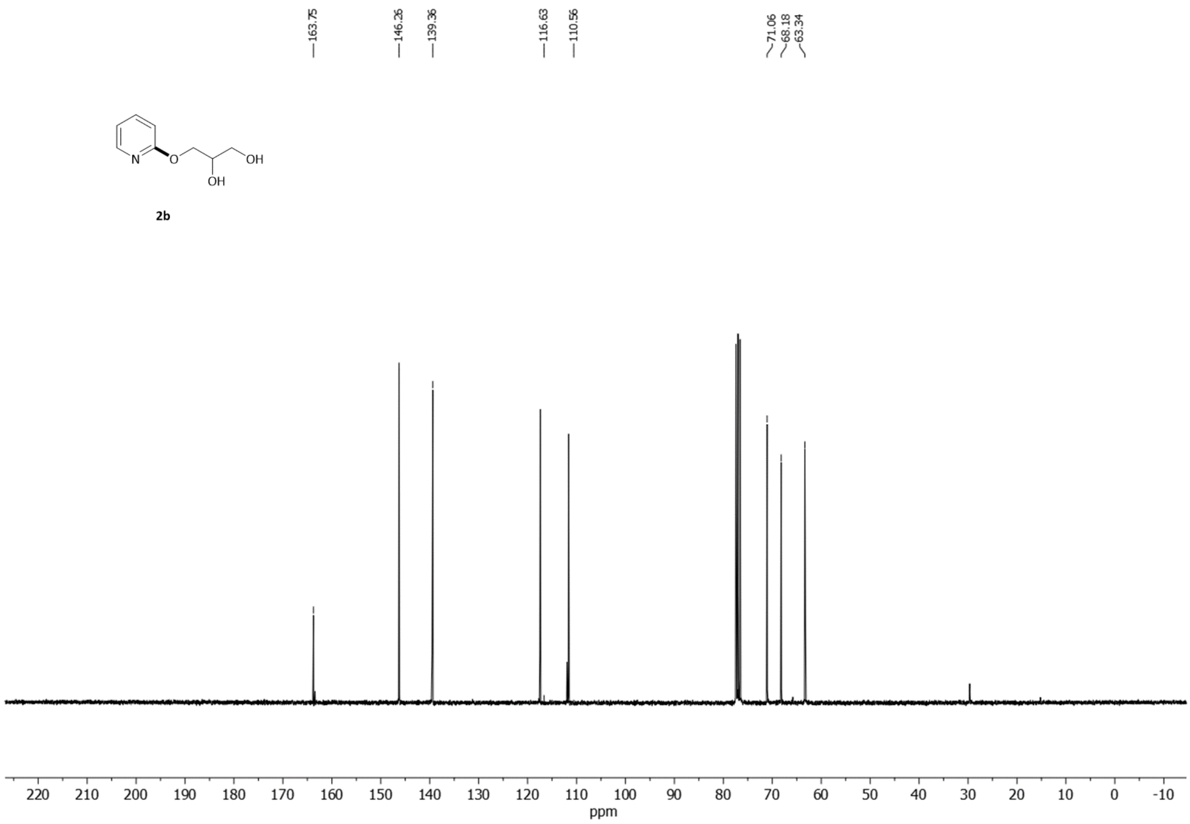

$^{1}H$ NMR, 600 MHz, $CDCl_3$

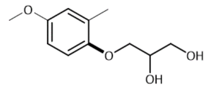

**2c**

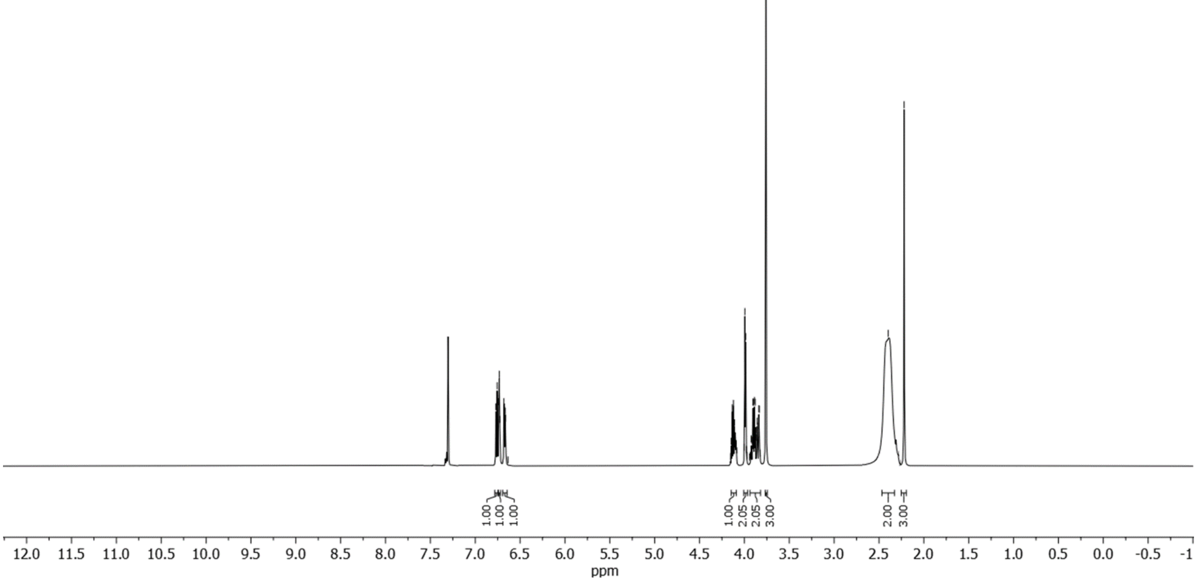


$^{13}C$ NMR, 150 MHz, $CDCl_3$

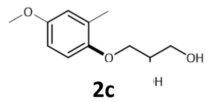

**2c**

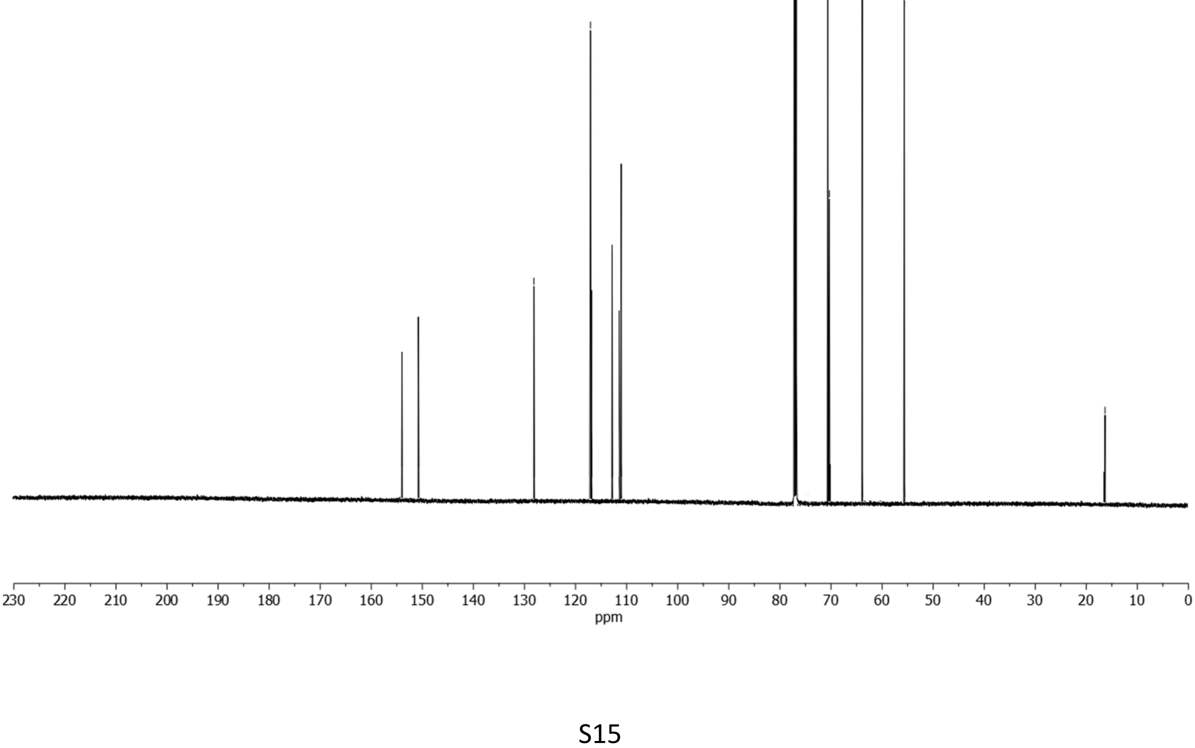

$^1H$ NMR, 600 MHz, $CDCl_3$

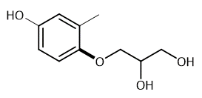

**2d**

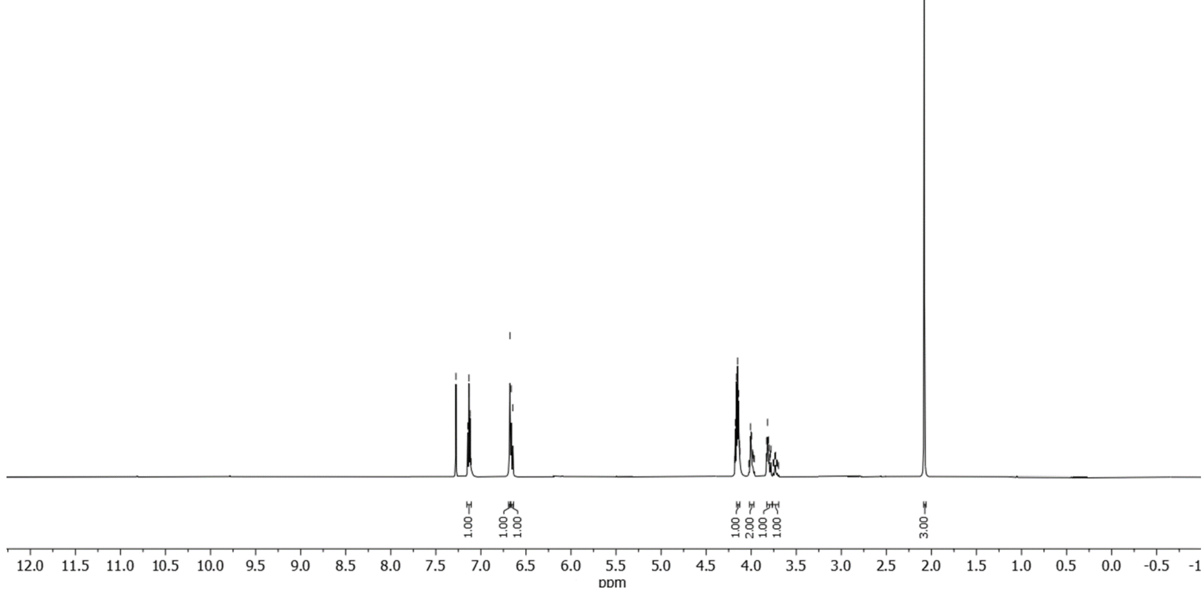


$^{13}C$ NMR, 150 MHz, $CDCl_3$

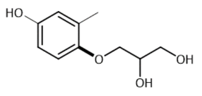

**2d**

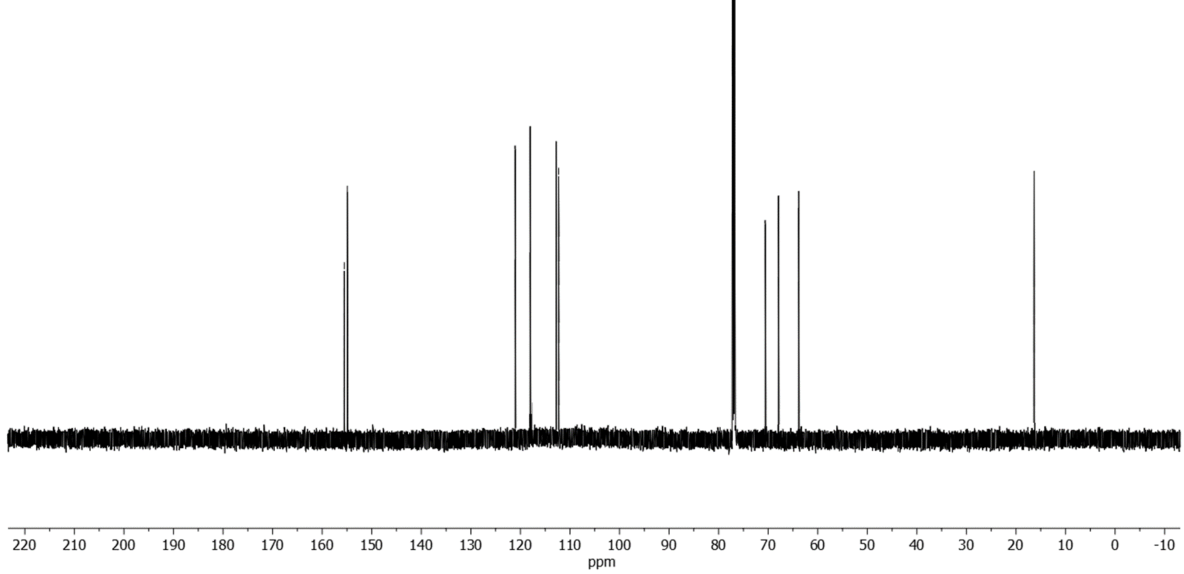

$^{1}H$ NMR, 600 MHz, $CDCl_3$

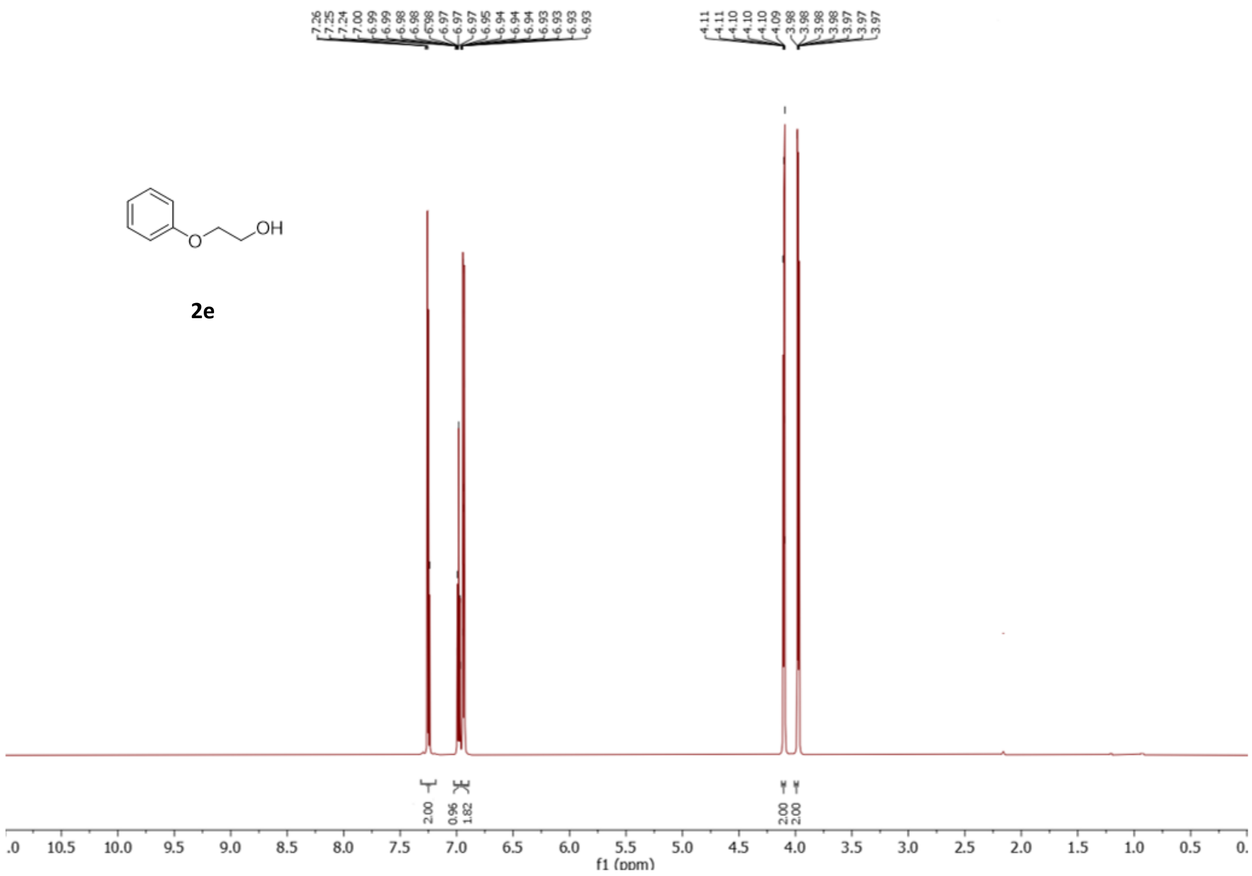


$^{13}C$ NMR, 150 MHz, $CDCl_3$

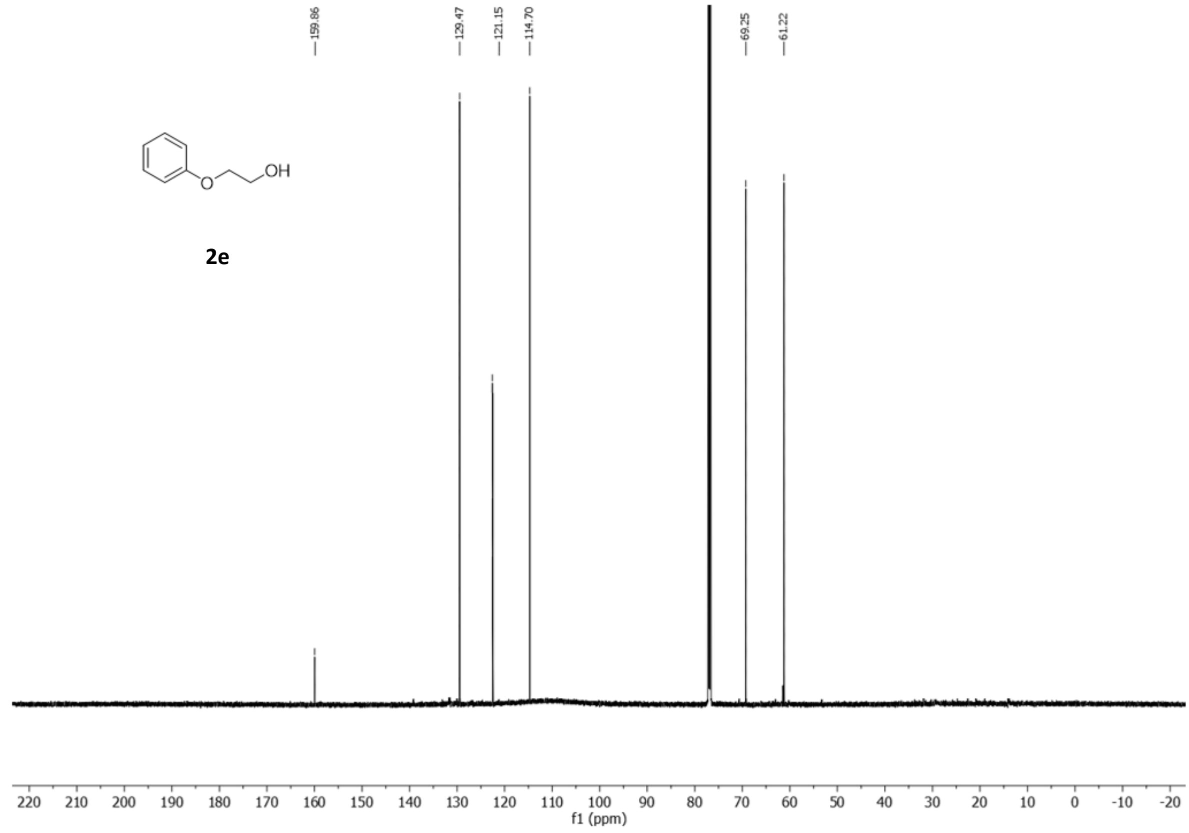

$^{1}H$ NMR, 600 MHz, $CDCl_3$

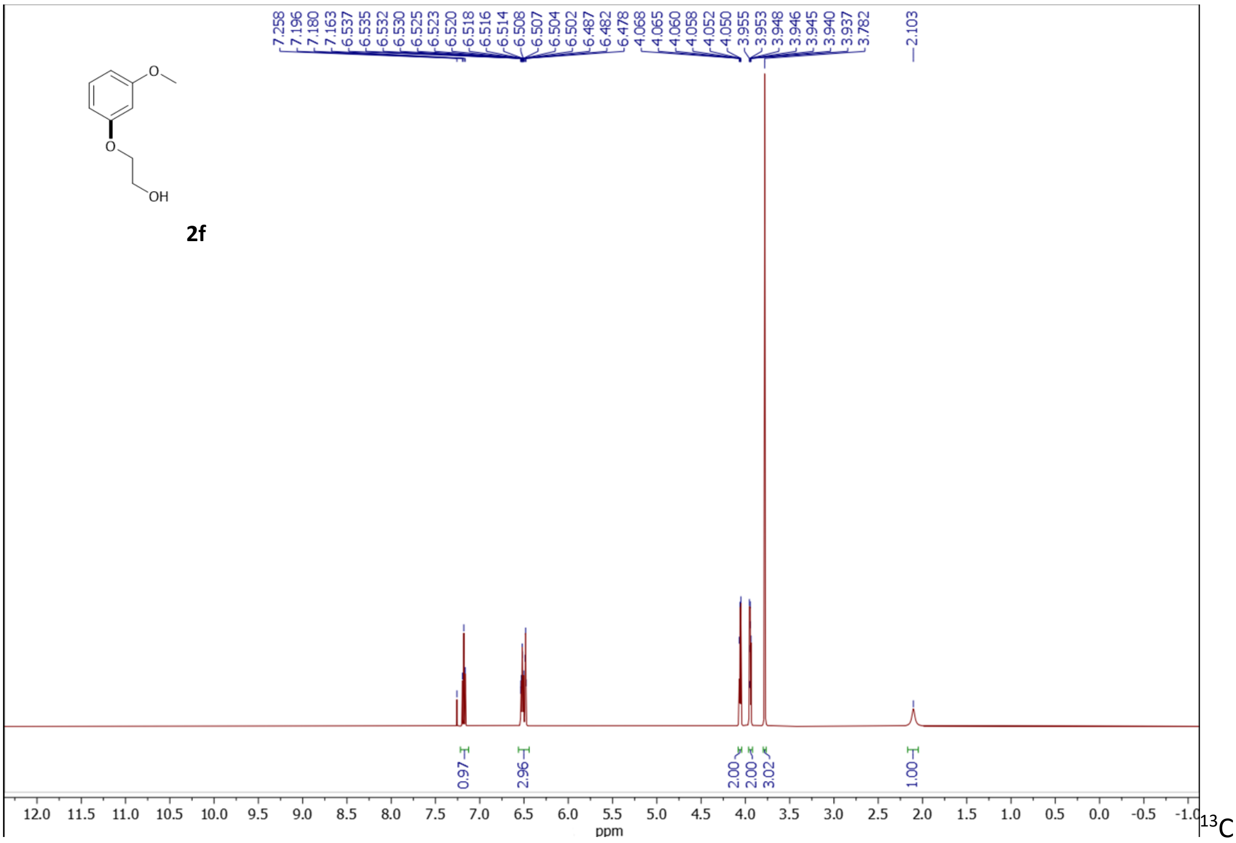


$^{13}C$ NMR, 150 MHz, $CDCl_3$

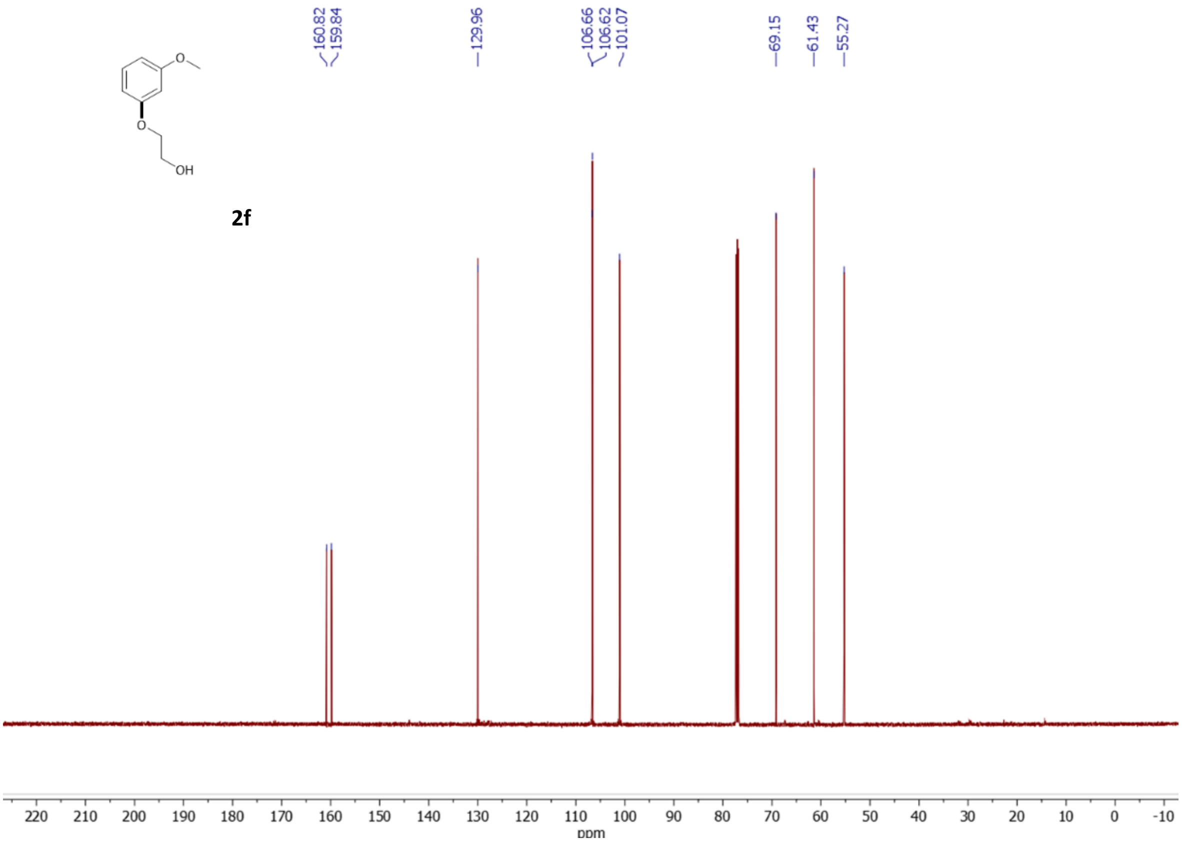

$^1$H NMR, 600 MHz, $CDCl_3$

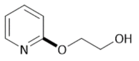


**2g**

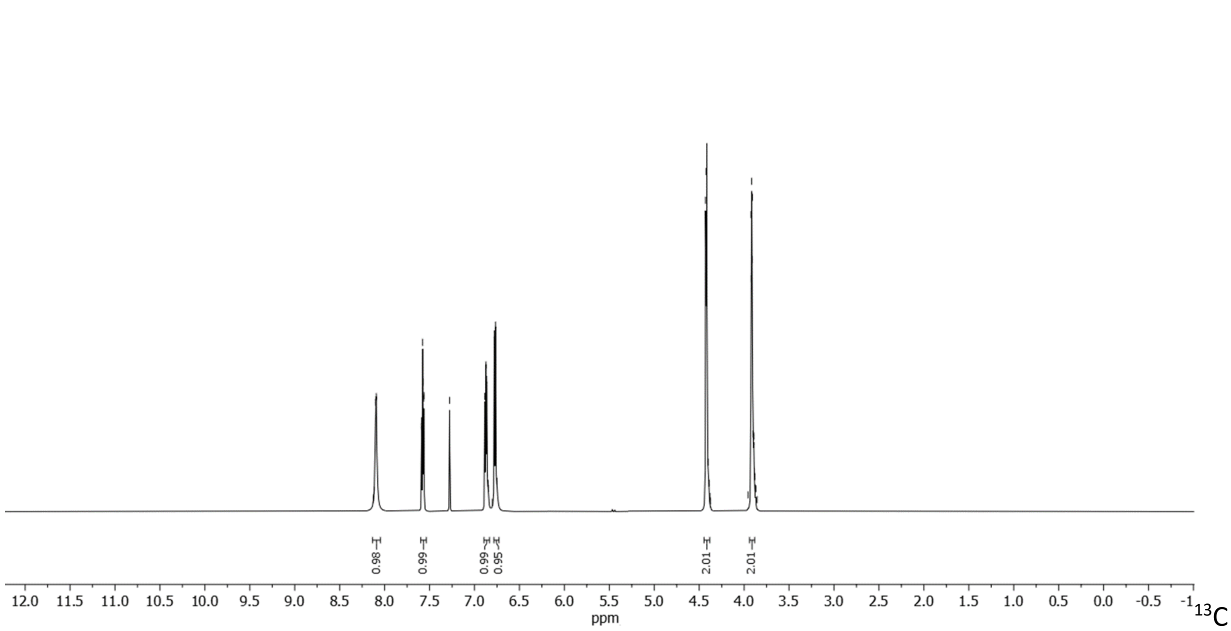


$^{13}$C NMR, 150 MHz, $CDCl_3$

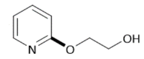


**2g**

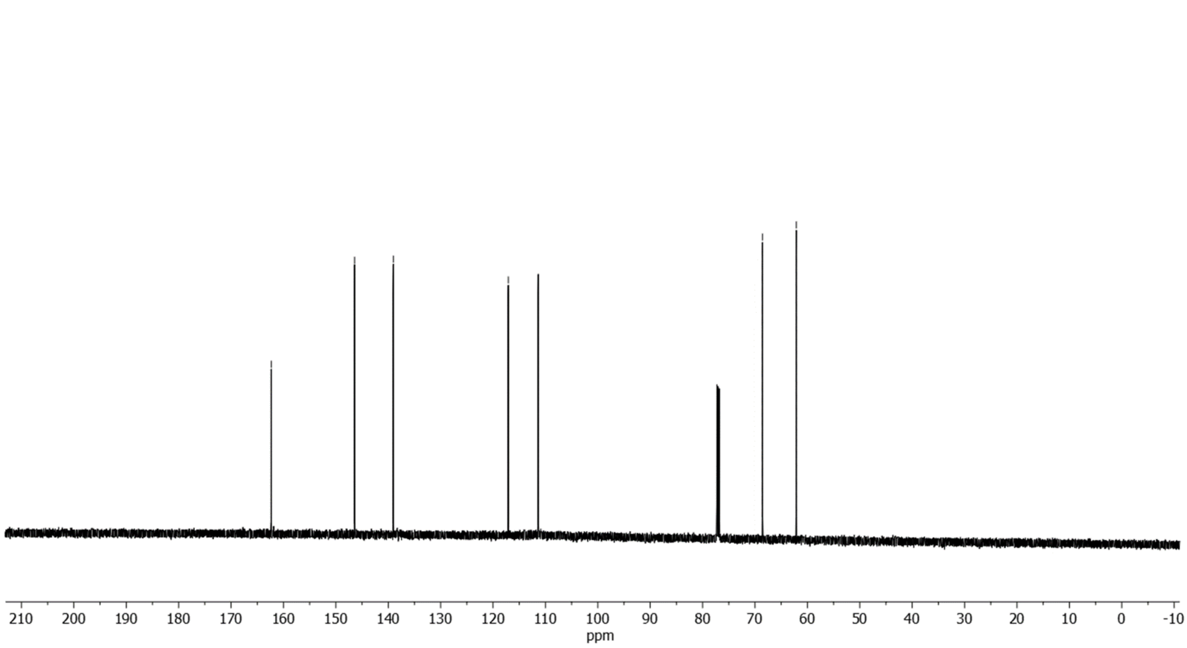

$^{1}H$ NMR, 600 MHz, $CDCl_3$

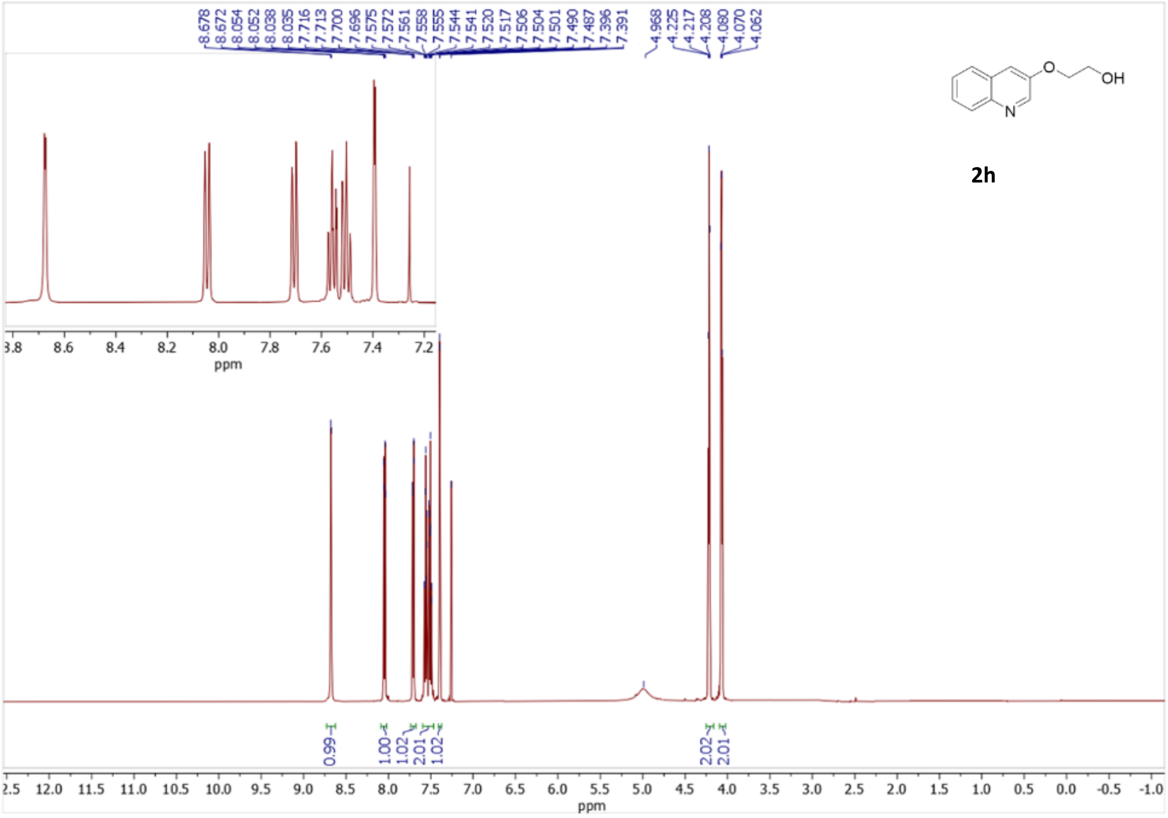


$^{13}C$ NMR, 150 MHz, $CDCl_3$

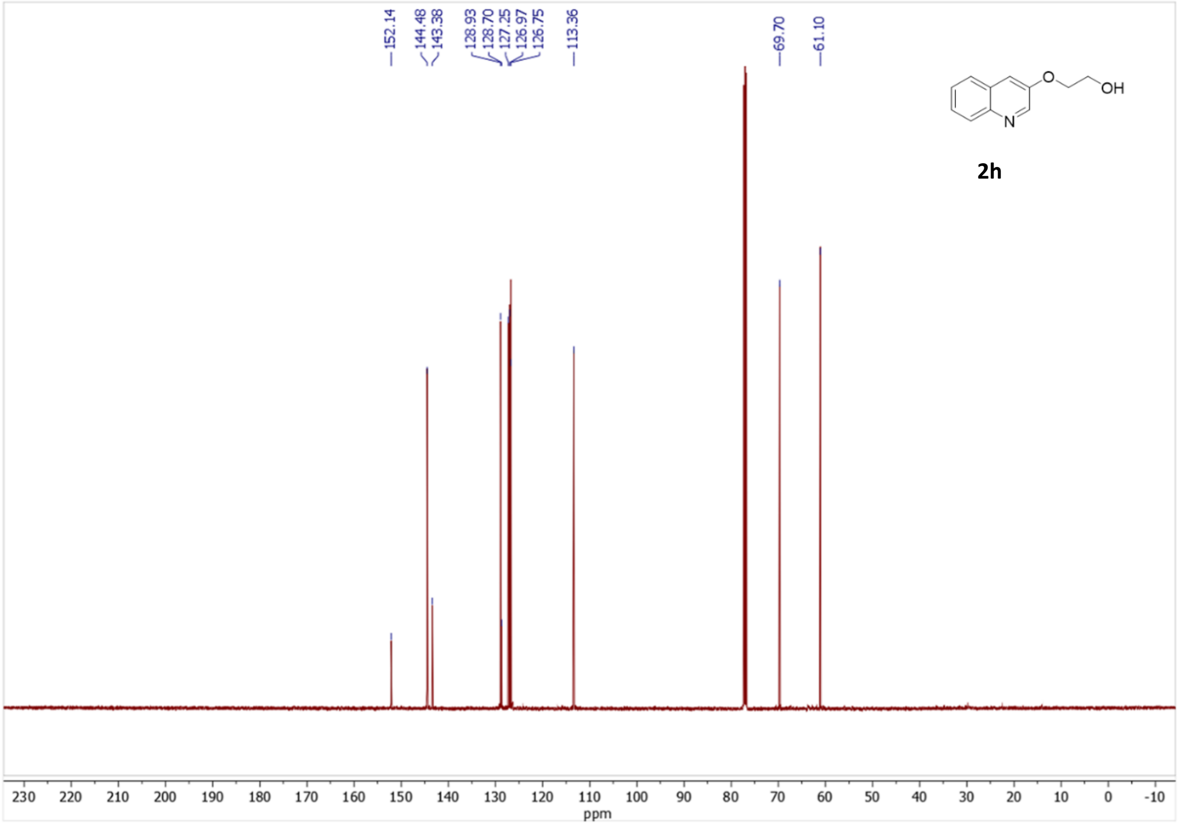

$^{1}H$ NMR, 600 MHz, $CDCl_3$

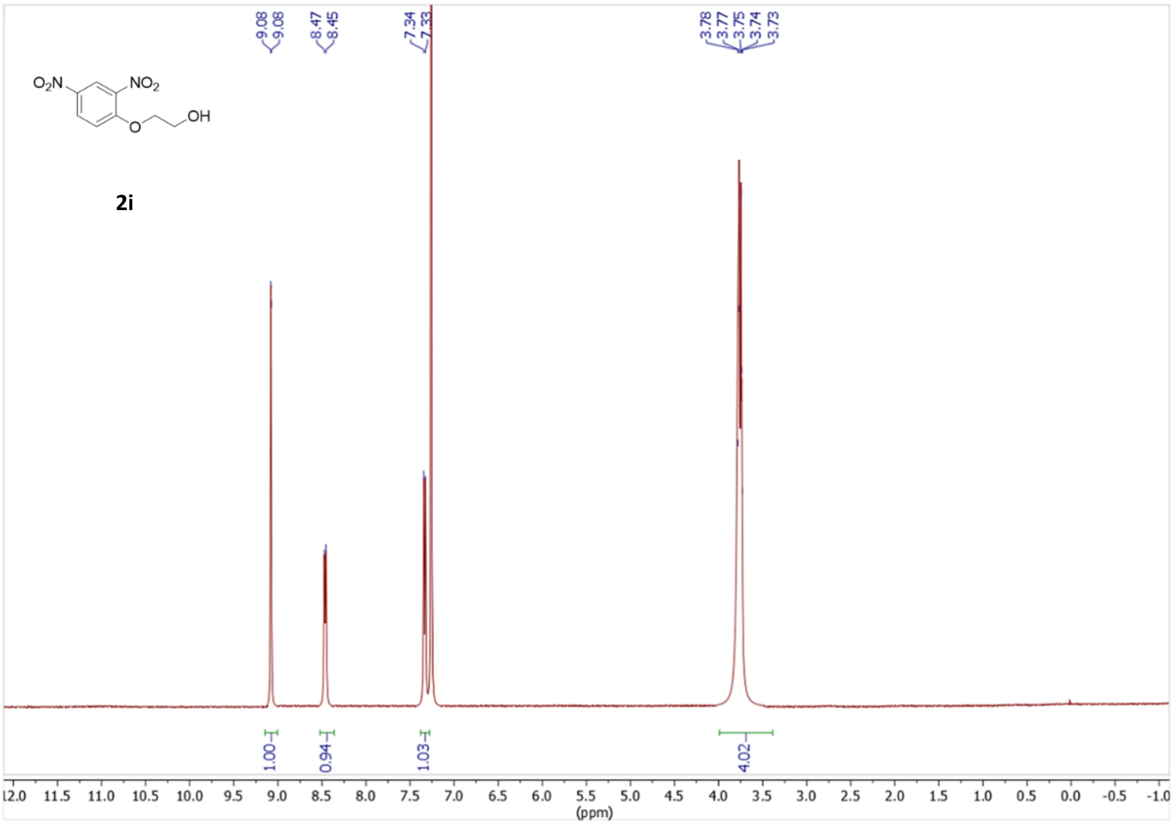


$^{13}C$ NMR, 150 MHz, $CDCl_3$

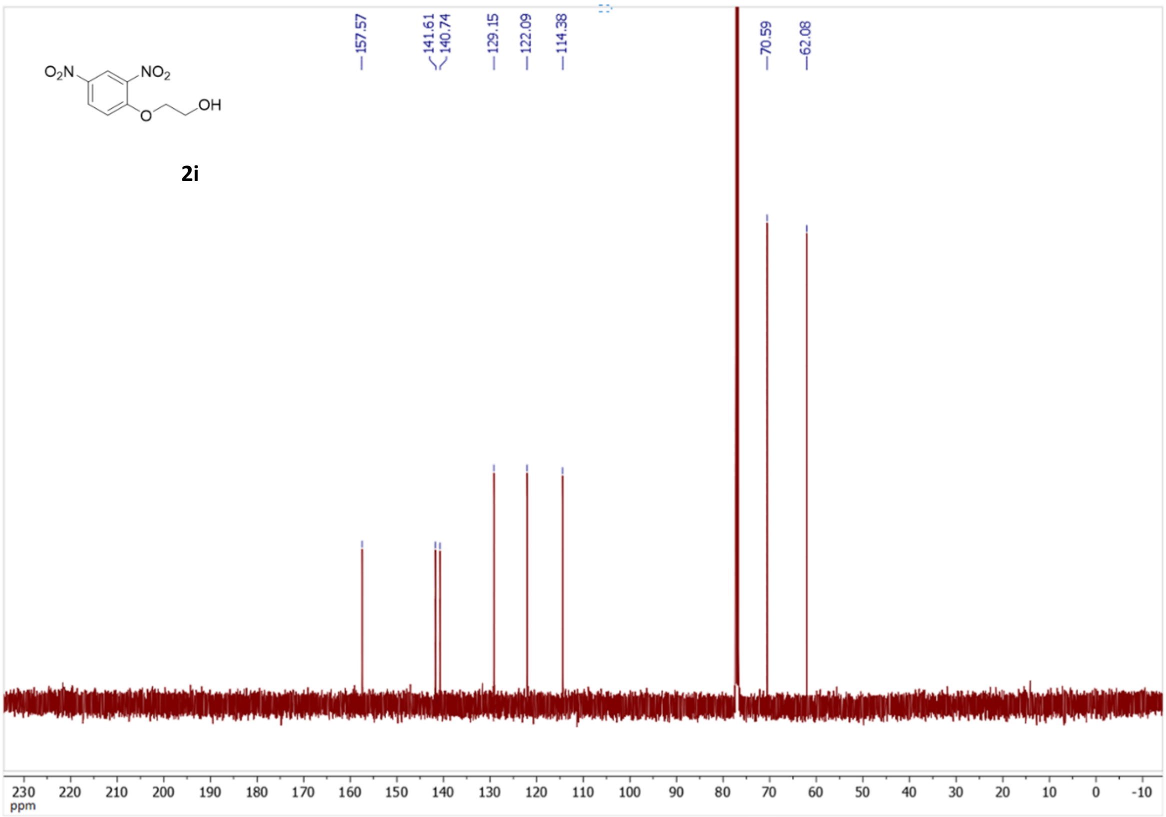

$^{1}H$ NMR, 600 MHz, $CDCl_3$

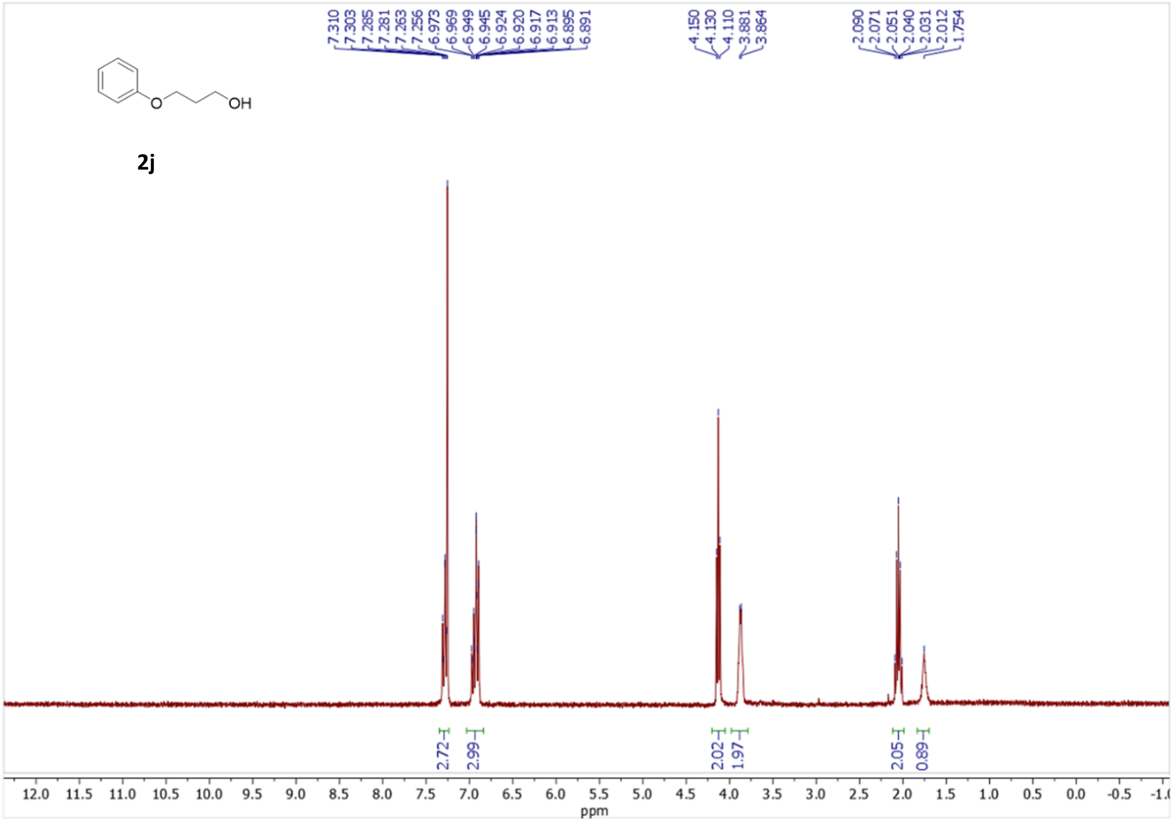


$^{13}C$ NMR, 150 MHz, $CDCl_3$

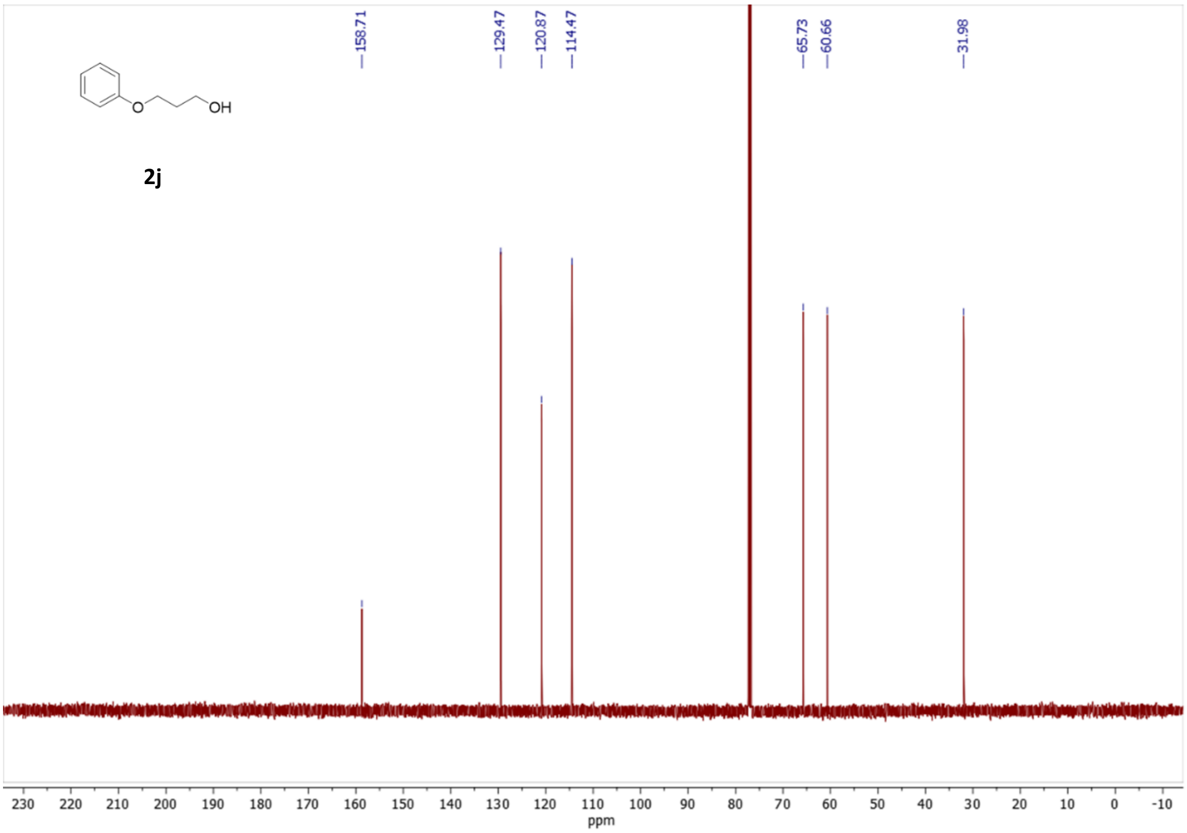

$^{1}$H NMR, 600 MHz, CDCl$_3$

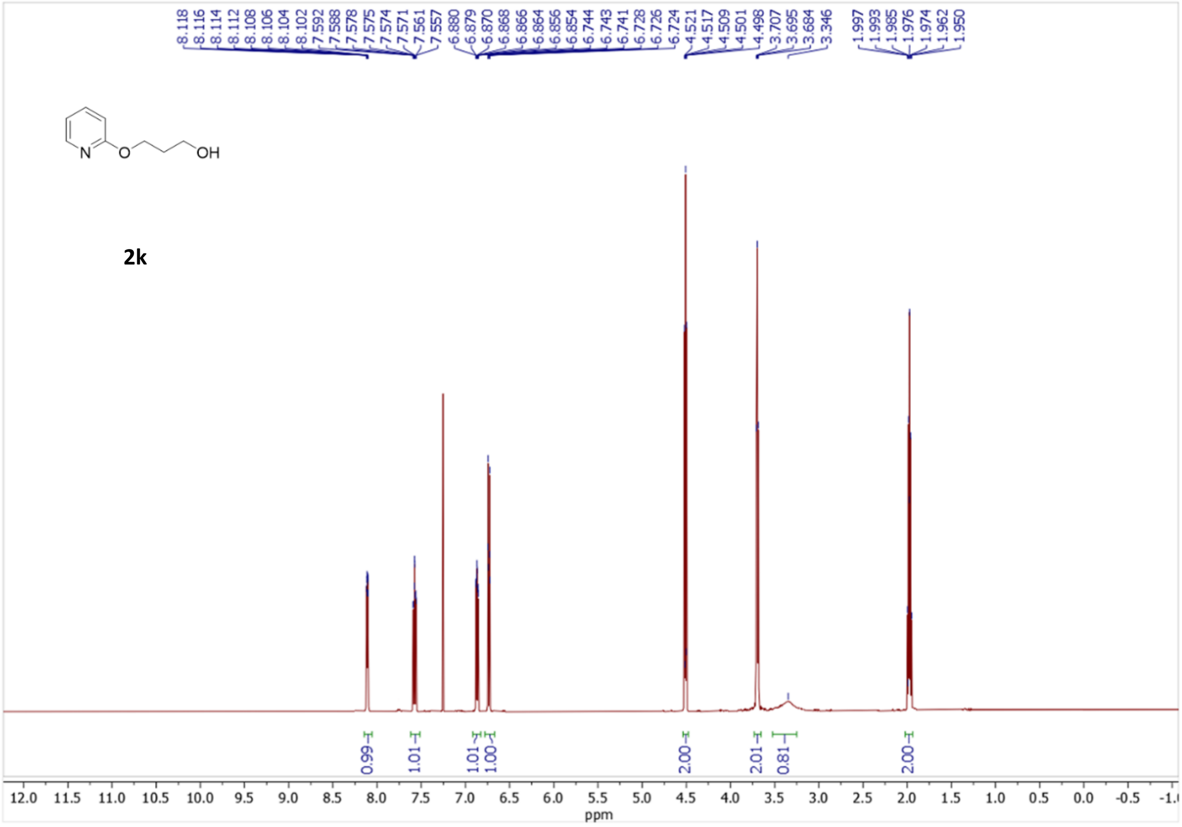


$^{13}$C NMR, 150 MHz, CDCl$_3$

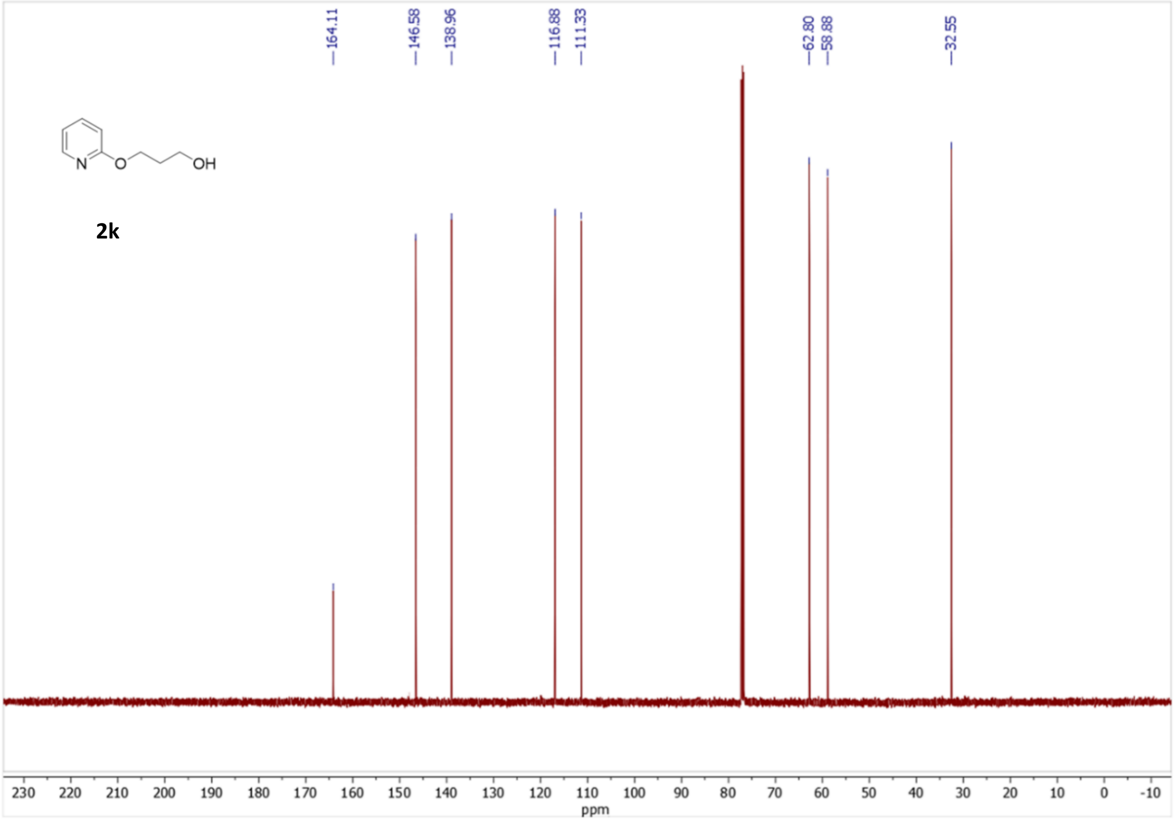

$^1$H NMR, 600 MHz, $CDCl_3$

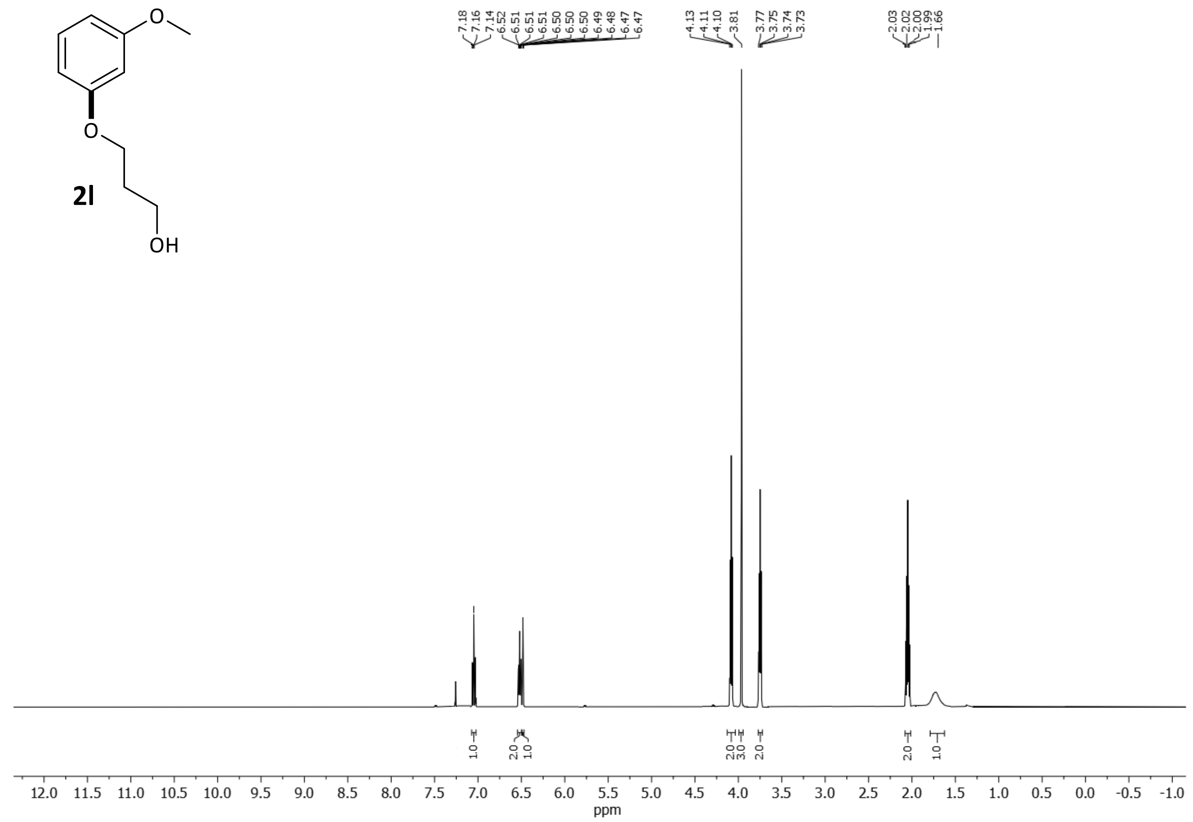


$^{13}$C NMR, 150 MHz, $CDCl_3$

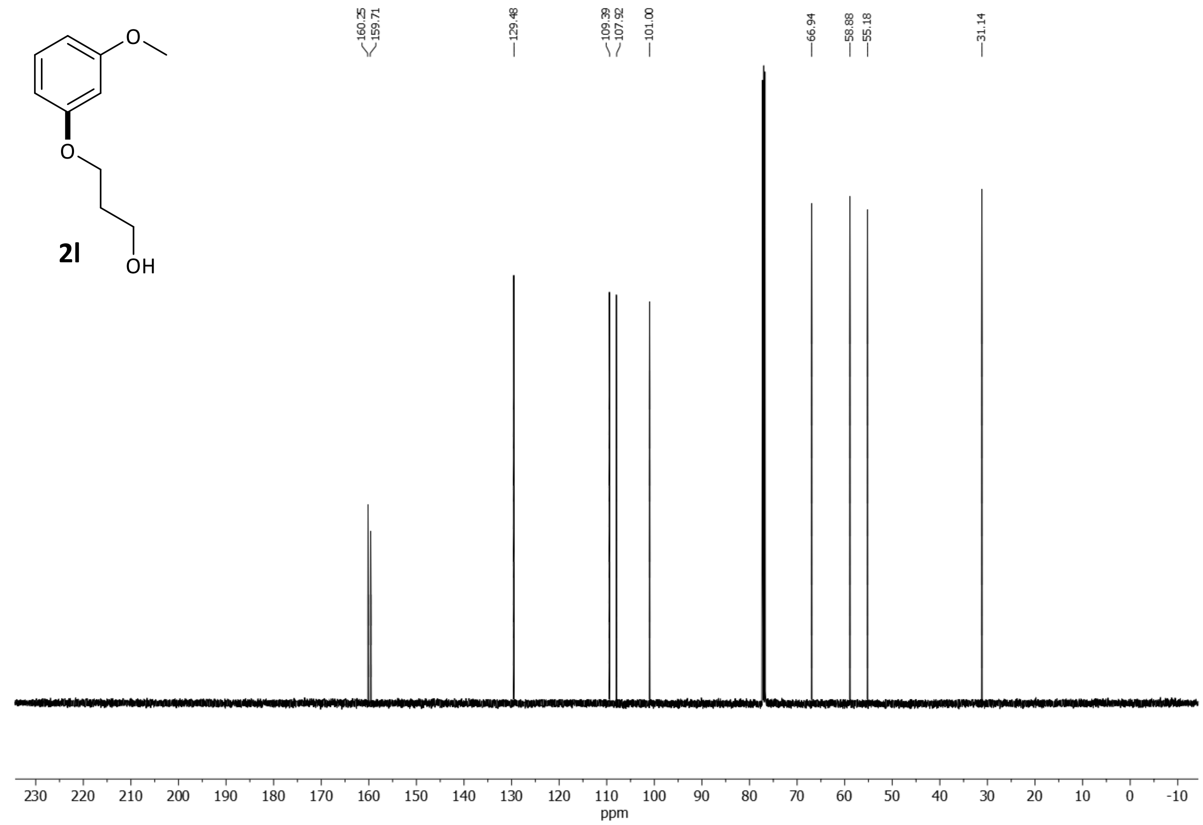

$^{1}H$ NMR, 600 MHz, $CDCl_3$

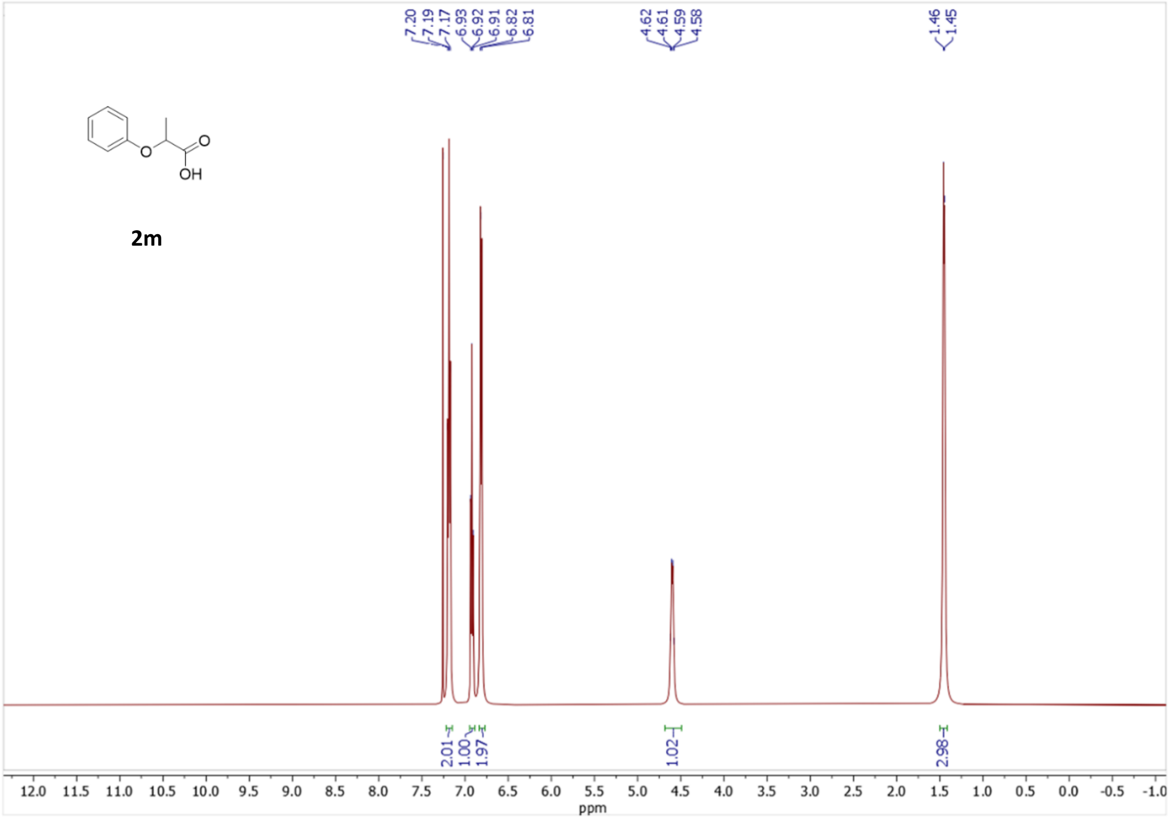


$^{13}C$ NMR, 150 MHz, $CDCl_3$

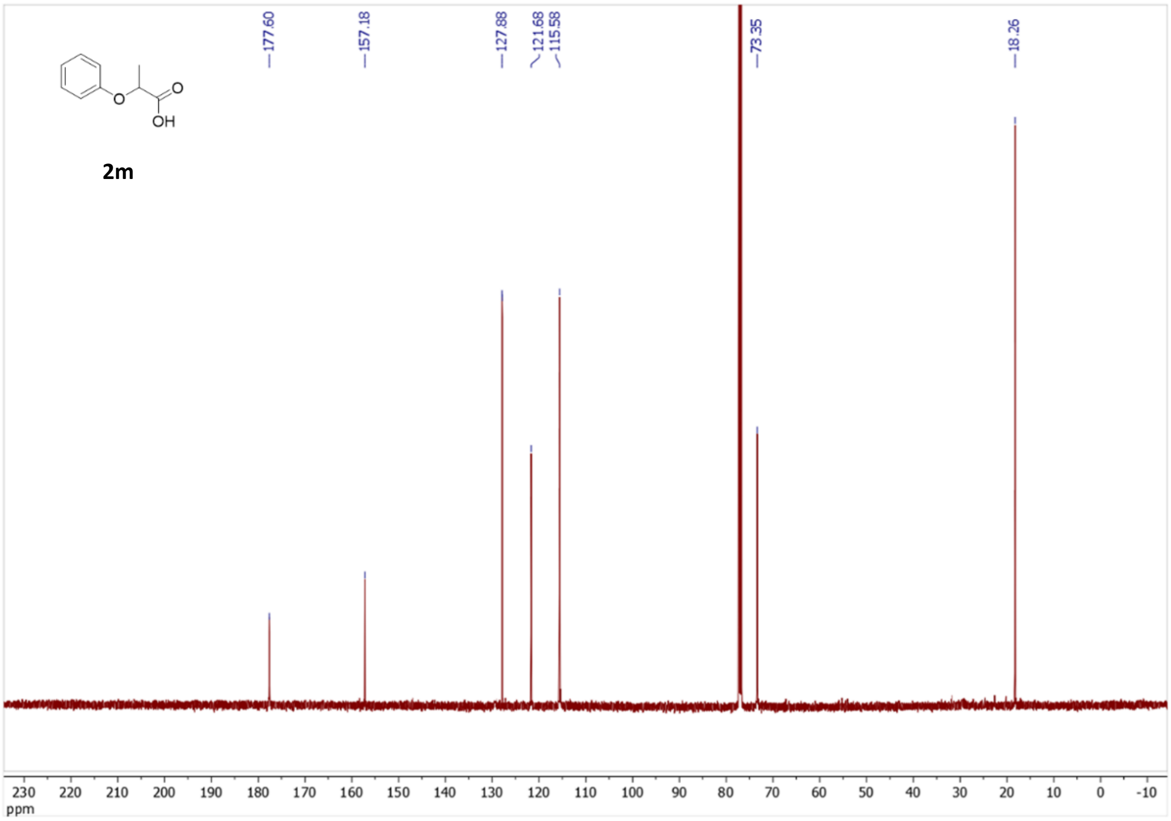

$^{1}H$ NMR, 600 MHz, $CDCl_3$

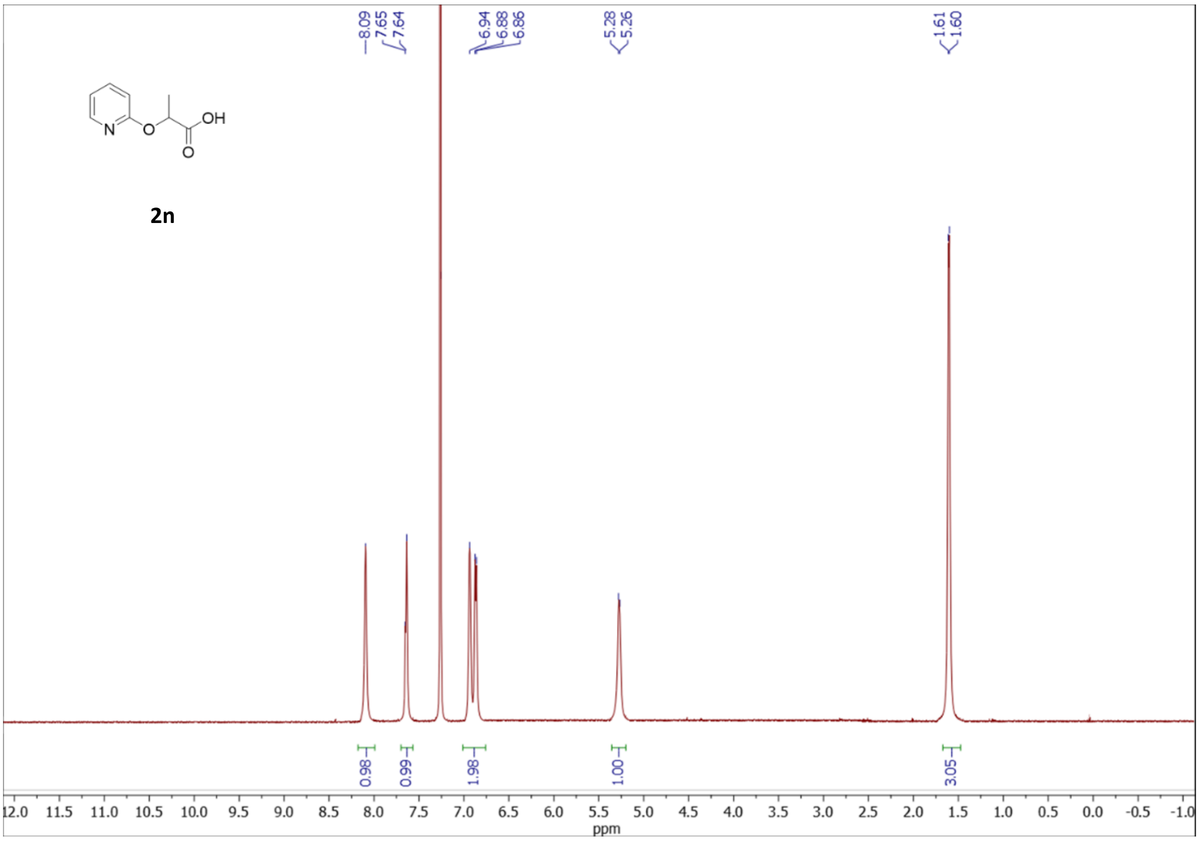


$^{13}C$ NMR, 150 MHz, $CDCl_3$

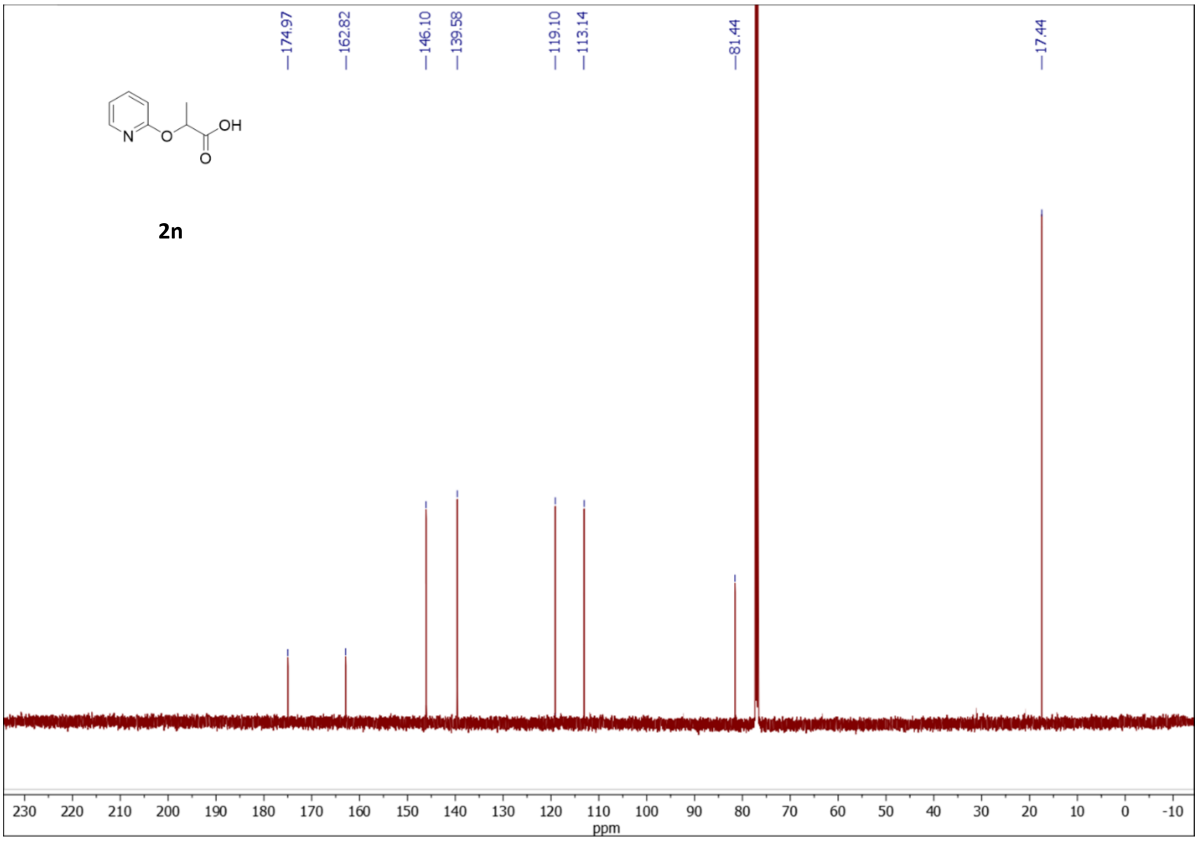

$^{1}H$ NMR, 600 MHz, $CDCl_3$

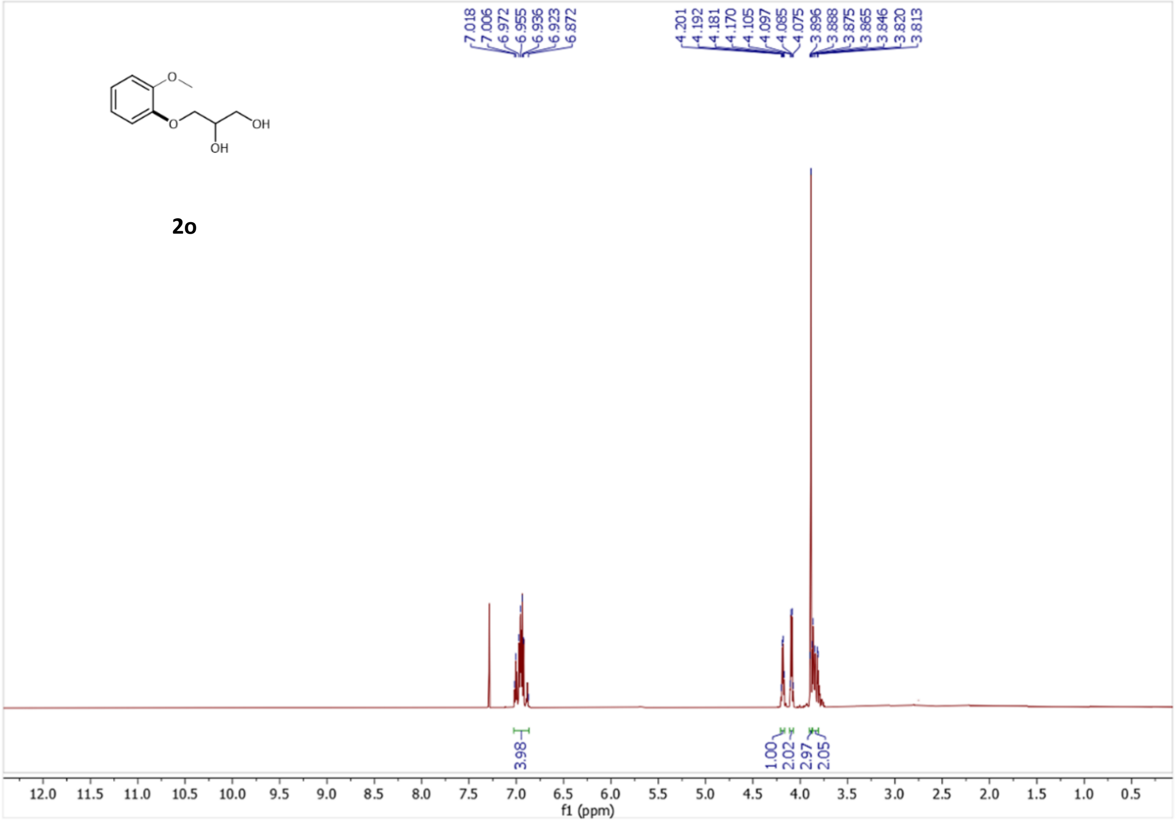


$^{13}C$ NMR, 150 MHz, $CDCl_3$

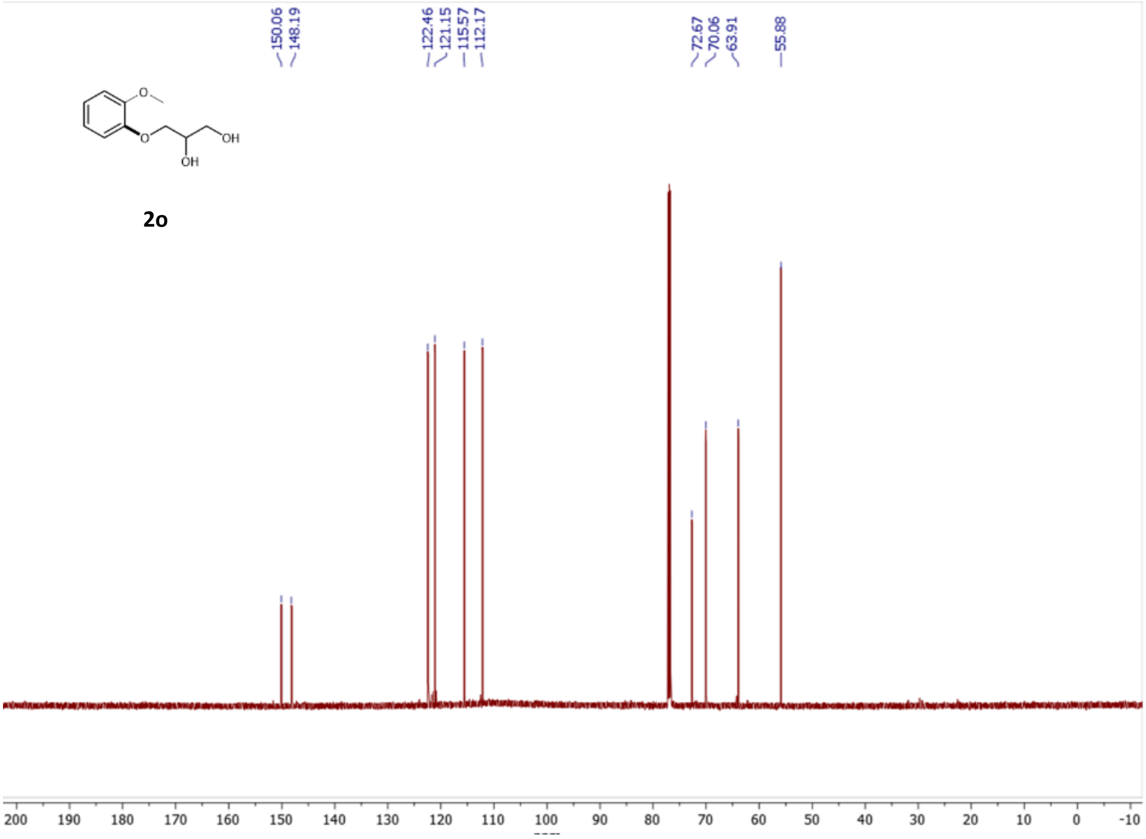

$^{1}$H NMR, 600 MHz, $CDCl_3$

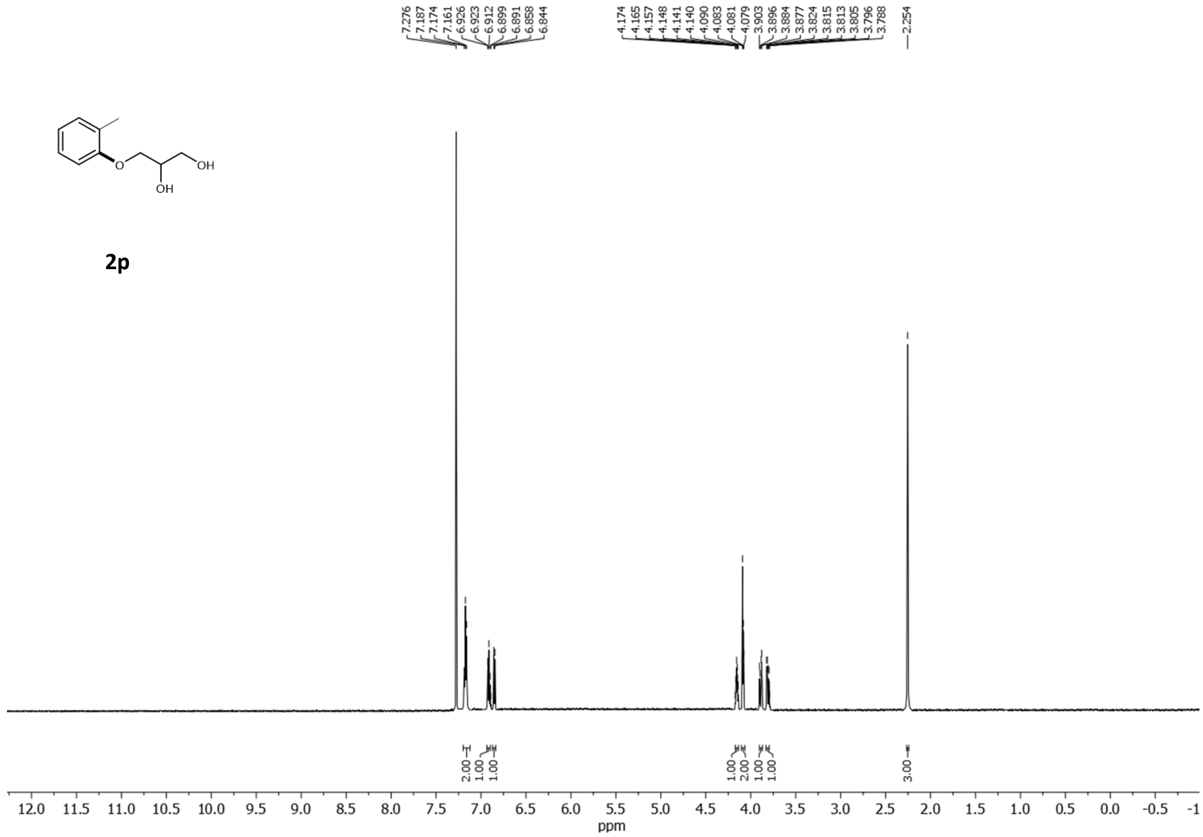

**2p**

$^{13}$C NMR, 150 MHz, $CDCl_3$

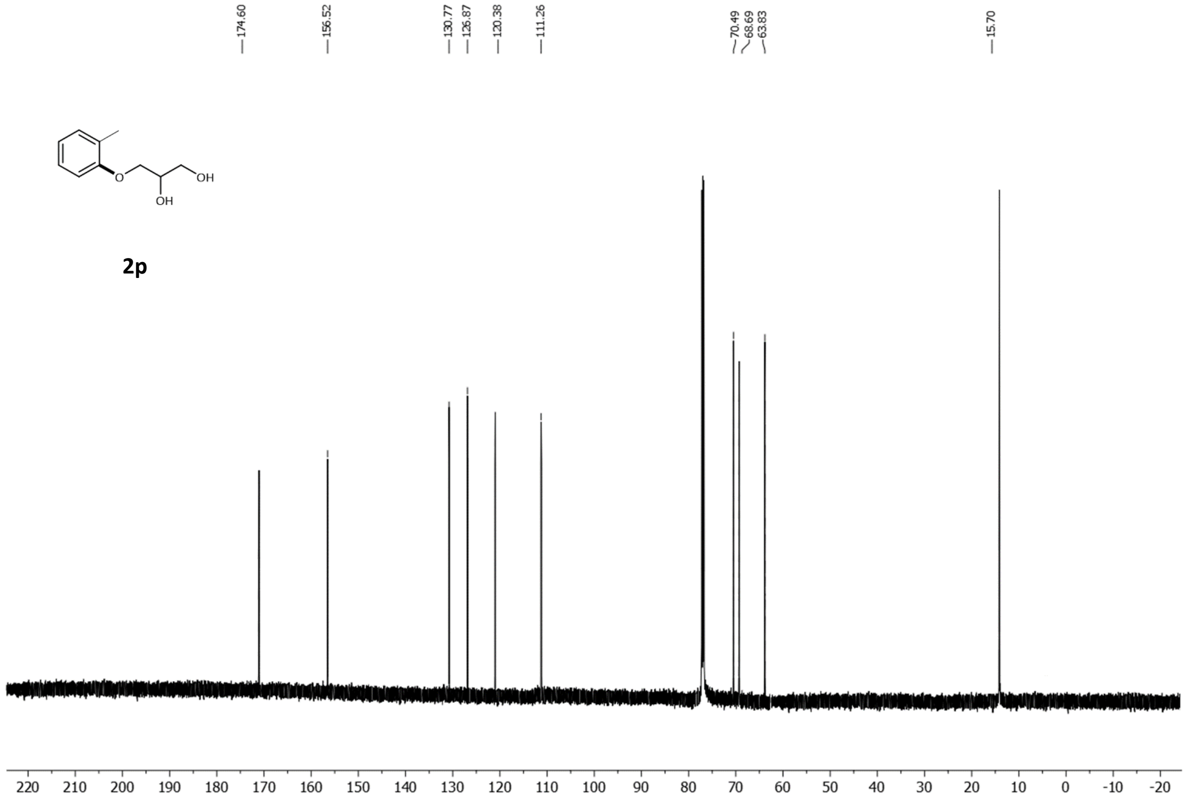

**2p**

$^{1}H$ NMR, 600 MHz, $CDCl_3$

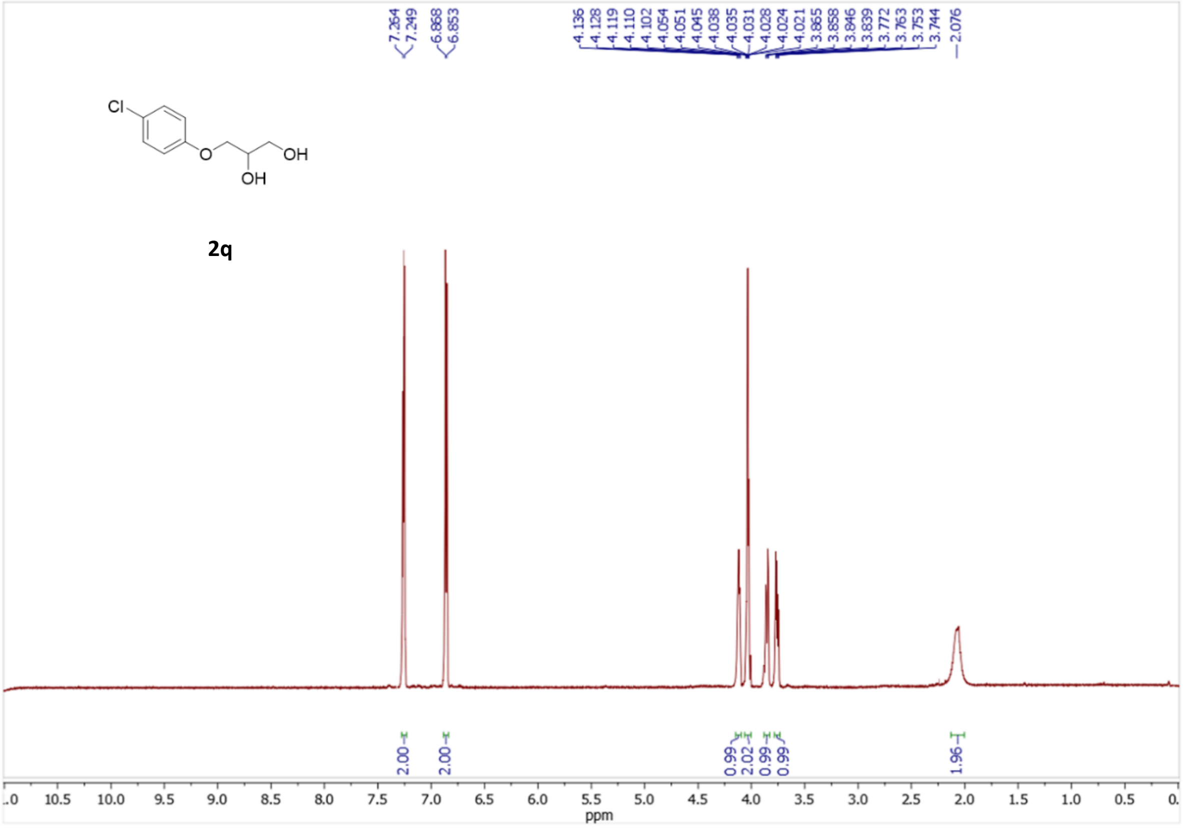


$^{13}C$ NMR, 150 MHz, $CDCl_3$

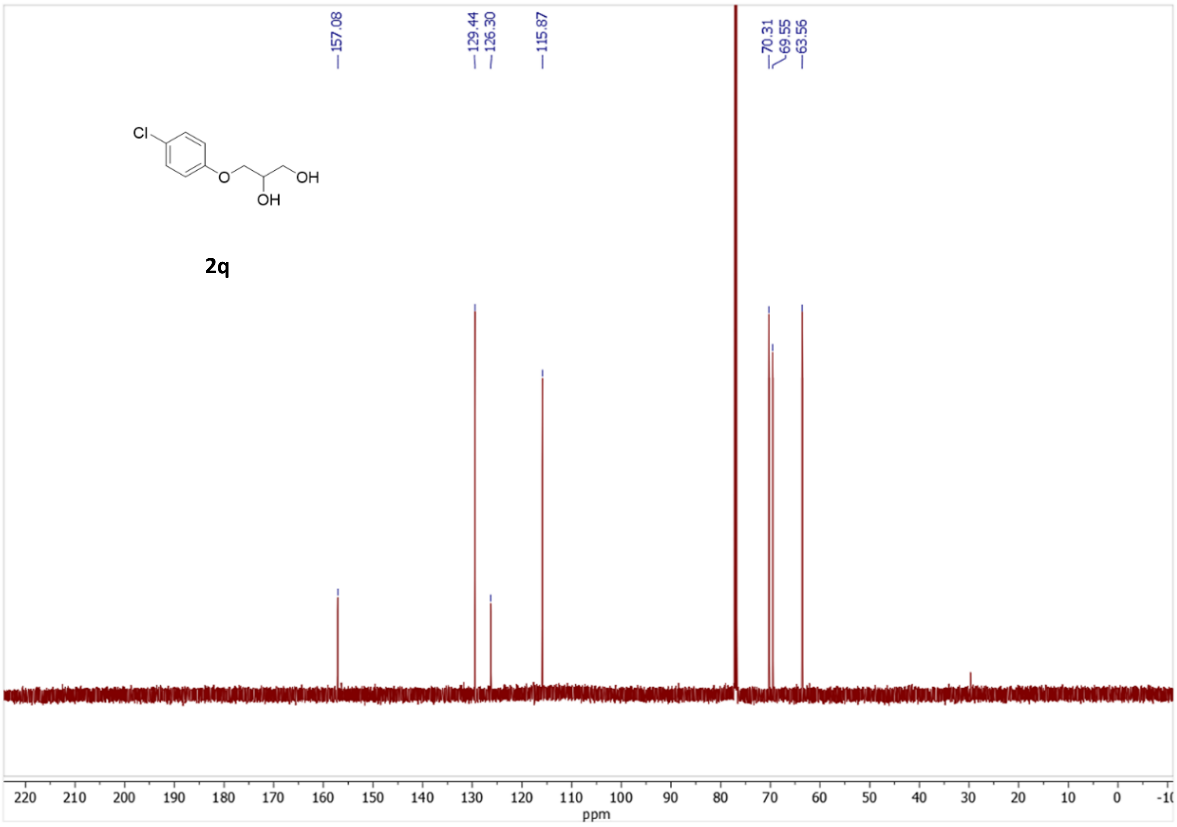

Supplement: Supplementary file 1 — Supporting Information [file CSSC-15-0-s001.pdf]
